# Extending real‐time MRI of the oral cavity using simultaneous multislice and compressed sensing

**DOI:** 10.1002/mrm.70085

**Published:** 2025-09-15

**Authors:** Isaac Watson, Mike Angus, Elisa Zamboni, David Mitchell, Angelika Sebald, Aneurin J. Kennerley

**Affiliations:** ^1^ Biomedical Imaging Science Department, Leeds Institute of Cardiovascular and Metabolic Medicine University of Leeds Leeds United Kingdom; ^2^ School of Physics, Engineering & Technology University of York York United Kingdom; ^3^ School of Psychology University of Nottingham Nottingham United Kingdom; ^4^ York Cross‐disciplinary Centre for Systems Analysis University of York York United Kingdom; ^5^ Department of Chemistry University of York York United Kingdom; ^6^ Department of Sports & Exercise Science Manchester Metropolitan University Manchester United Kingdom

**Keywords:** compressed sensing, dynamic imaging, radial sampling, real‐time MRI, simultaneous multislice

## Abstract

**Purpose:**

To demonstrate a real‐time MRI (rtMRI) sequence that can image multiple slices simultaneously and apply them to image the dynamics of the oral cavity. Specifically, we demonstrate the imaging of tongue movement, speech, and swallowing.

**Methods:**

We developed a radial rtMRI sequence with multiband excitation. Two sampling schemes were explored: a golden‐angle trajectory and a golden‐angle trajectory adapted for simultaneous multislice acceleration. Additionally, we developed a compressed sensing reconstruction pipeline. Phantom and in vivo rtMRI data were acquired on a 3 T system using a standard head/neck coil.

**Results:**

We show that the proposed technique can acquire rtMRI videos at a range of temporal resolutions (up to 25 ms). Example applications of tongue movement, speech, and swallowing are shown. Additionally, we show that the sequence is robust to changes in slice distance and coil compression level.

**Conclusion:**

Combining rtMRI with multiband excitation and compressed sensing reconstruction enables imaging of the oral cavity at high (up to 25 ms) temporal resolution.

## 1 INTRODUCTION

Real‐time MRI (rtMRI), combined with conventional structural MRI investigations, provides enhanced diagnostic capability by enabling the noninvasive imaging of free movement without the need for any gating or synchronization equipment.^1^ Currently, the main application of rtMRI is monitoring cardiac motion.^2^ However, it also enables the recording of simple nonrepetitive joint motion^3^ and the rich dynamics of the oral cavity such as speech and swallowing.^4^, ^5^, ^6^

rtMRI techniques typically work by exploiting both highly undersampled non‐Cartesian trajectories (e.g., radial and spiral trajectories) and sequences that enable short TRs (typically FLASH or balanced steady‐state free precession).^2^ The requirement of a short TR prevents interleaved acquisition of slices, limiting the anatomical coverage (in a single scan) of such sequences. To mitigate this limitation, the simultaneous multi‐slice (SMS) acceleration technique can be implemented. SMS acceleration uses specially designed RF pulses (superposition of multiple frequency bands) to excite multiple slices simultaneously during slice gradient application.^7^, ^8^, ^9^ The slices are then separated using reconstruction techniques that exploit the spatial redundancy present in array‐based receiver coils.

SMS is now routinely used with EPI sequences to reduce scan time and/or enable a lower TR in functional MRI.^10^ It can also be combined with non‐Cartesian sampling for dynamic imaging applications.^11^, ^12^, ^13^, ^14^ For example, previous work by Yang et al. used multiband excitation with a variable density spiral sequence to perform cardiac perfusion imaging (with electrocardiogram gating).^11^ Recent work by Yagiz et al. introduced a spiral SMS sequence with compressed sensing reconstruction designed for real‐time cardiac imaging applications at low field strengths.^15^ However, the long spiral readout limits its application in areas prone to off‐resonance effect (particularly at high field strengths), such as the oral cavity. Radial SMS sequences have also been used in cardiac perfusion and cine imaging applications.^12^, ^13^ Sequences employing SMS acceleration typically require a separate set of single‐slice calibration scans (known as *prescans*) to calculate the coil sensitivity profiles required for reconstruction.^8^ This results in an increase in the overall scan time and may pose problems when imaging movement because the reference scans will not align with the subsequent imaging data.

Calibration‐less SMS sequences overcome this problem by estimating the coil sensitivity profiles from the acquired data,^14^ removing the need for prescans. This has been demonstrated in cardiac and T_1_‐mapping applications.^14^, ^16^ In this work, we present a calibration‐less SMS rtMRI sequence that uses radial sampling and a compressed sensing reconstruction pipeline. We compare two radial sampling schemes: a standard golden angle (GA) trajectory and a GA trajectory optimized from improved SMS reconstruction. The use of GA sampling schemes enables the arbitrary selection of temporal resolution.^17^, ^18^

We specifically focus on the function of oral cavity as an application of the proposed method. This is a challenging area to image because motion (e.g., as speech or swallowing) is complex and nonperiodic; thus, gated techniques or assumptions about periodicity cannot be used. rtMRI does not rely on any assumptions regarding periodicity. In addition, the temporal and spatial resolution must be sufficient to capture the motion of fast articulators; Lingala et al. recommended a minimum temporal resolution of 70 ms and a minimum spatial resolution of 3.5 mm^2^ for imaging vowel/consonant formation.^4^ An additional challenge is that in the oral cavity there are multiple air–tissue and tissue–tissue boundaries distributed over a relatively small volume. This can result in significant off‐resonance artifacts, particularly when employing complex sampling schemes such as non‐Cartesian trajectories. Finally, in the oral cavity, the anatomy that enables motion is spread out across the entire volume. In this circumstance, simultaneous multislice rtMRI techniques can enable improved coverage of the function of the oral cavity with no loss of temporal resolution. We show that the developed sequence can record the dynamics of the oral cavity at high temporal (up to 25 ms) and spatial resolutions (2.2mm^2^) and concurrently image up to three slices simultaneously. Compared to single‐slice rtMRI, the proposed methodology provides a fuller overview of function, particularly when imaging nonsynchronous motions involving multiple anatomical structures. We show results in both a dynamic phantom and in vivo in healthy volunteers at a range of undersampling factors and slice distances.

## 2 METHODS

All data was acquired on a 3 T Magnetom Vida system (software version XA31A; Siemens Healthineers, Erlangen) using a 64‐channel head/neck receive coil. To test the sequence, a dynamic phantom (see section 2.1) was constructed. In vivo imaging data was then obtained from two healthy volunteers. One volunteer performed a tongue mobility task in which they extended and then retracted their tongue; additionally, they also performed an empty swallow (i.e., swallowing saliva rather than a bolus of food/liquid). The second volunteer performed a speech task in which they pronounced all English vowels. Ethical approval for all scanning was provided by Manchester Metropolitan University (Manchester, UK).

For all tasks, 20 s of data is recorded and up to five slices are simultaneously acquired. The imaging parameters used were TR =2.5 ms, TE =1.4 ms, flip angle $=5^{{}^{\circ}}$, points per spokes =128, bandwidth = 1447 Hz/pixel, FOV = 280×280 mm^2^, slice thickness = 8 mm.

### 2.1 Sequence

**FIGURE 1:**
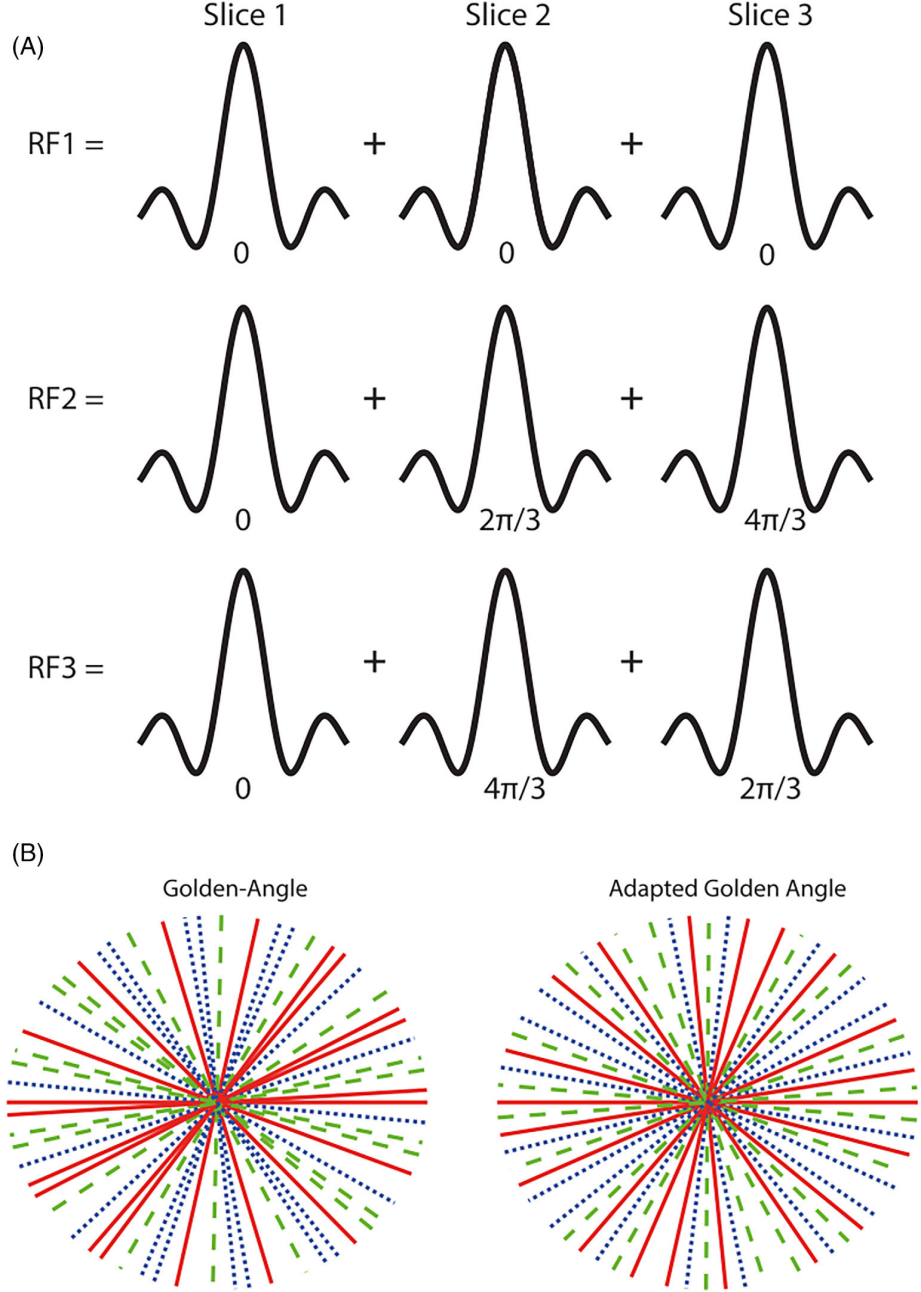
(A) Demonstration (for three slices) of the phase cycling scheme used to generate the RF pulses required for the sequence. (B) Comparison of GA (left) and SMS GA (right) sampling schemes, showing the SMS GA trajectory creates a more even distribution of the RF pulses. This improves the destructive interference between slices. The sampling patterns shown are for a frame consisting of 25 spokes and three slices acquired simultaneously. The red solid lines correspond to the first RF pulse; green dashed lines correspond to the second RF pulse; and the blue dotted lines correspond to the third RF pulse. GA, golden‐angle; SMS, simultaneous multi‐slice.

An RF‐spoiled radial FLASH sequence was used to acquire rtMRI data. Transverse magnetization was spoiled using a random RF spoiling scheme.^19^ The sequence was altered to excite multiple ($N_{\mathrm{Sli}}$) slices simultaneously through use of multiband RF pulses generated on the scanner. To improve slice separation, a controlled aliasing in parallel imaging results in higher acceleration (CAIPIRINHA)–phase modulation scheme was used.^9^, ^20^ A number of pulses equal to the number of slices are generated (Figure 1, top). For pulse n, the phase difference, $\varphi_{n}$, between the summed single‐band pulses is cycled following Equation (1).

(1) $$\varphi_{n}=\mathrm{mod}\left[\frac{2\pi }{N_{\mathrm{Sli}}}\cdot n,2\pi \right].$$

Two radial trajectories are used in this work (Figure 1, bottom). The first is a GA radial trajectory in which the angular gap θ between subsequent radial spokes is given by Equation (2), where GR is the golden ratio.^17^, ^18^

(2) $$\mathrm{GR}=\frac{180}{\mathrm{GR}}\;\approx 111.25^{{}^{\circ}}.$$

The second trajectory, which we refer to as SMS GA, was proposed by Wu et al. and is designed to improve destructive interference between slices (although a direct comparison between GA and SMS GA was not performed in their work).^20^ In SMS GA, the angular increment between spokes is given by Equation (3).

(3) $$\theta =\frac{180}{\mathrm{GR}\cdot N_{\mathrm{Sli}}}\approx \frac{111.25}{N_{\mathrm{Sli}}}.$$

In both trajectories, the total number of spokes acquired was chosen such that the desired acquisition time covers the period of the movement. The spokes were then retrospectively binned to achieve the desired temporal resolution (i.e., number of spokes/frame).

### 2.2 Reconstruction

**FIGURE 2:**
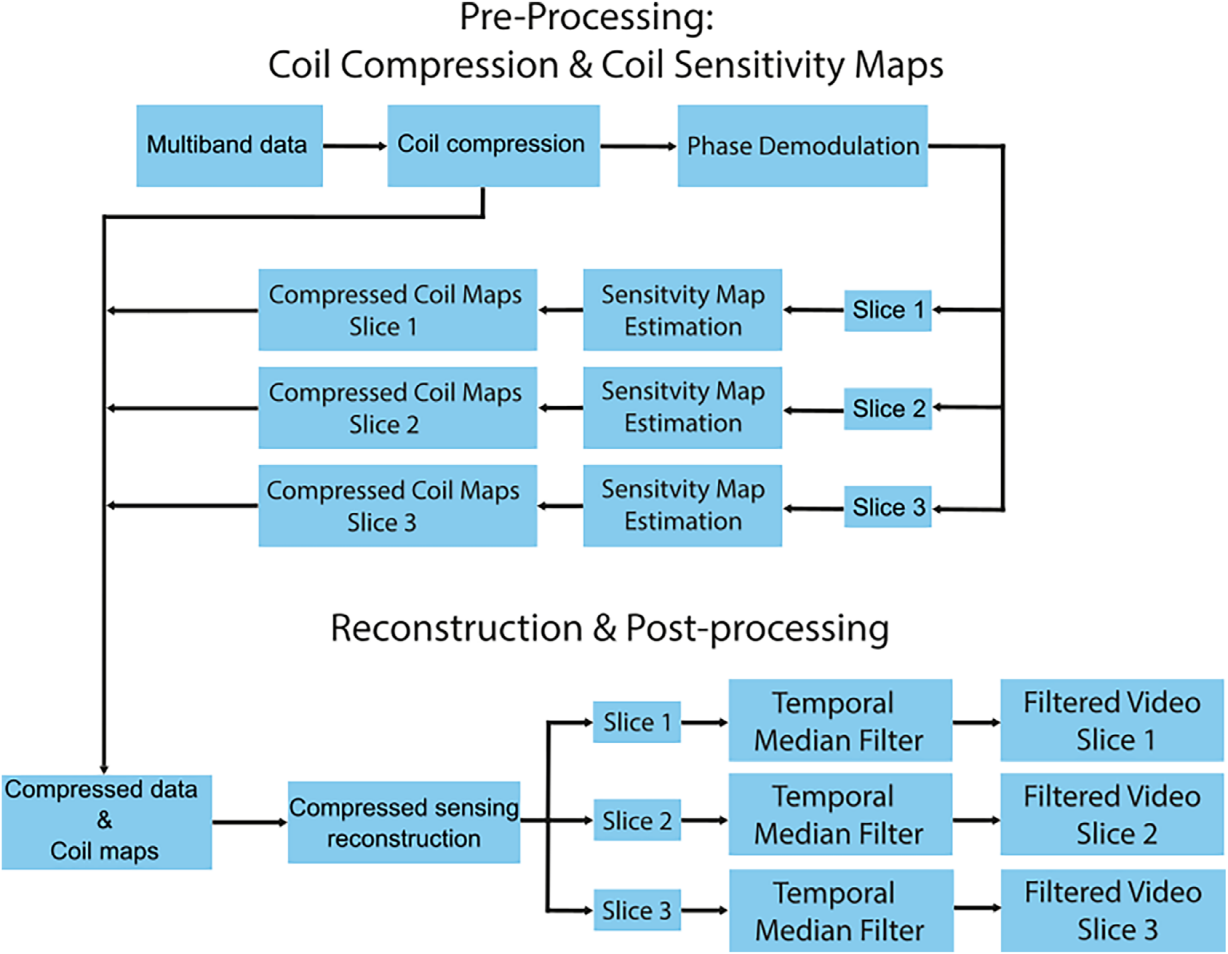
Diagram of the reconstruction pipeline used in this work. First, the data is compressed and coil sensitivity maps are calculated for each slice. Using compressed data and coil sensitivity maps, the rtMRI videos are reconstructed using a compressed sensing reconstruction algorithm. All steps are performed offline in MatLab R2023b. rtMRI, real‐time MRI.

A four‐step reconstruction pipeline is used in this work (Figure 2). We employ finite temporal difference regularization, which has been used extensively in dynamic radial imaging.^18^, ^20^ Our reconstruction pipeline is similar to that developed by Feng et al. but with adaptions to account for the additional RF phase modulation.^21^ All reconstruction is performed offline in MatLab R2023b (MathWorks, Natick, MA) on a desktop computer (AMD Ryzen 7700x 8 Core CPU, 64GB DDR5 memory). A GPU implementation of a nonuniform fast Fourier transform (NUFFT) algorithm was implemented (using CUDA C) to reduce reconstruction time.^22^ All other reconstruction steps were performed on the CPU (with no multithreading).

#### 2.2.1 Data preprocessing

To reduce computation time, the multiband rtMRI data is reduced to a set of 16 virtual channels using a principle component analysis (PCA) channel compression algorithm.^23^ The effect of coil compression on reconstruction time and image quality is shown in section 3.4.

We used a pipeline similar to that proposed by Wang et al. to calculate coil sensitivity profiles.^12^ The entire time‐series of coil compressed k‐space was used for coil sensitivity estimation. The coil‐compressed SMS radial k‐space data was multiplied with the complex conjugate of the phase cycling pattern. This results in a motion averaged k‐space for each slice. The k‐space for each slice was then transformed into the image domain, using the adjoint NUFFT, resulting in a set of coil images for each slice. Finally, to obtain the coil sensitivity profiles, FFT was applied to create a Cartesian k‐space; ESPIRIT was then used with the center region (24 × 24 points) of k‐space to recover coil sensitivity maps for each slice.^24^

#### 2.2.2 Image reconstruction and postprocessing

The reconstruction model used in this work is shown in Equation (4), where y is the multi‐channel k‐space data; $x_{i}$, $\varphi_{i}$, and $S_{i}$ are the images; CAIPIRINHA phase modulation and coil sensitivity maps for slice *i*, F is the NUFFT operator. The temporal finite difference transform, T, is used for regularization, with λ controlling the level of regularization.

(4) $$\hat{x}=\underset{x=\left(x_{1},x_{2},\ldots ,x_{\text{Nsli}}\right)}{\text{argmin}}\left[{\frac{1}{2}\left\|\sum_{i=1}^{\text{NSli}}\left(\varphi_{i}FS_{i}x_{i}\right)-y\right\|}_{2}^{2}+\lambda {\left\|Tx\right\|}_{1}\right].$$

The reconstruction problem is solved using the alternating direction method of multipliers (ADMM) algorithm with automated penalty parameter estimation.^25^, ^26^ After reconstruction, a temporal median filter (of width three frames) is applied to each slice to suppress artifacts caused by undersampling.^27^

The proposed reconstruction pipeline was compared against conjugate gradient (CG)‐SENSE, which does not use any form of regularization. This can be considered as using the proposed method with λ=0 (albeit with a different optimization algorithm).

#### 2.2.3 Regularization parameter selection

To find a suitable value for λ in Equation (4), a search over different values was performed using a three‐slice dataset (12 mm‐slice distance, 25 spokes/frame). Initially, a coarse set of values, increasing logarithmically from 0.0001 to 0.1, was used. This search was then repeated on a finer scale of 0.01 to 0.1 in steps of 0.01. A regularization value of λ (based on results shown in section 3.1) was used.

### 2.3 Dynamic phantom

A dynamic phantom designed to mimic the structure and movements of the oral cavity was designed, manufactured, and used to initially test the sequence. The model is composed of a tongue, mandible, and bilateral floor of the mouth and cheeks constructed from molded silicon (Smooth‐On Ecoflex 00–20 FAST, Macungie, PA) and filled with an agar hydrocolloid (Biozoon, Bremerhaven, Germany; concentration of 2 g per 100 mL of water). Anchor points are embedded into the phantoms structure. Nylon wire is attached to these points, which can be pulled to stretch and turn the model tongue. A full description of the design and construction of the phantom is shown in Text S1. Examples of how the phantom can be used to test the rtMRI sequence and perform investigations, such as examining the effect of metal surgical reconstruction plates, are also shown in Data S1.

## 3 RESULTS

Below, we present still images from a dynamic data series. Real‐time movies of the data (running at a range of temporal resolutions) are provided as supplementary material, and we recommend that the reader view these alongside the descriptions below.

### 3.1 Regularization parameter selection

**FIGURE 3:**
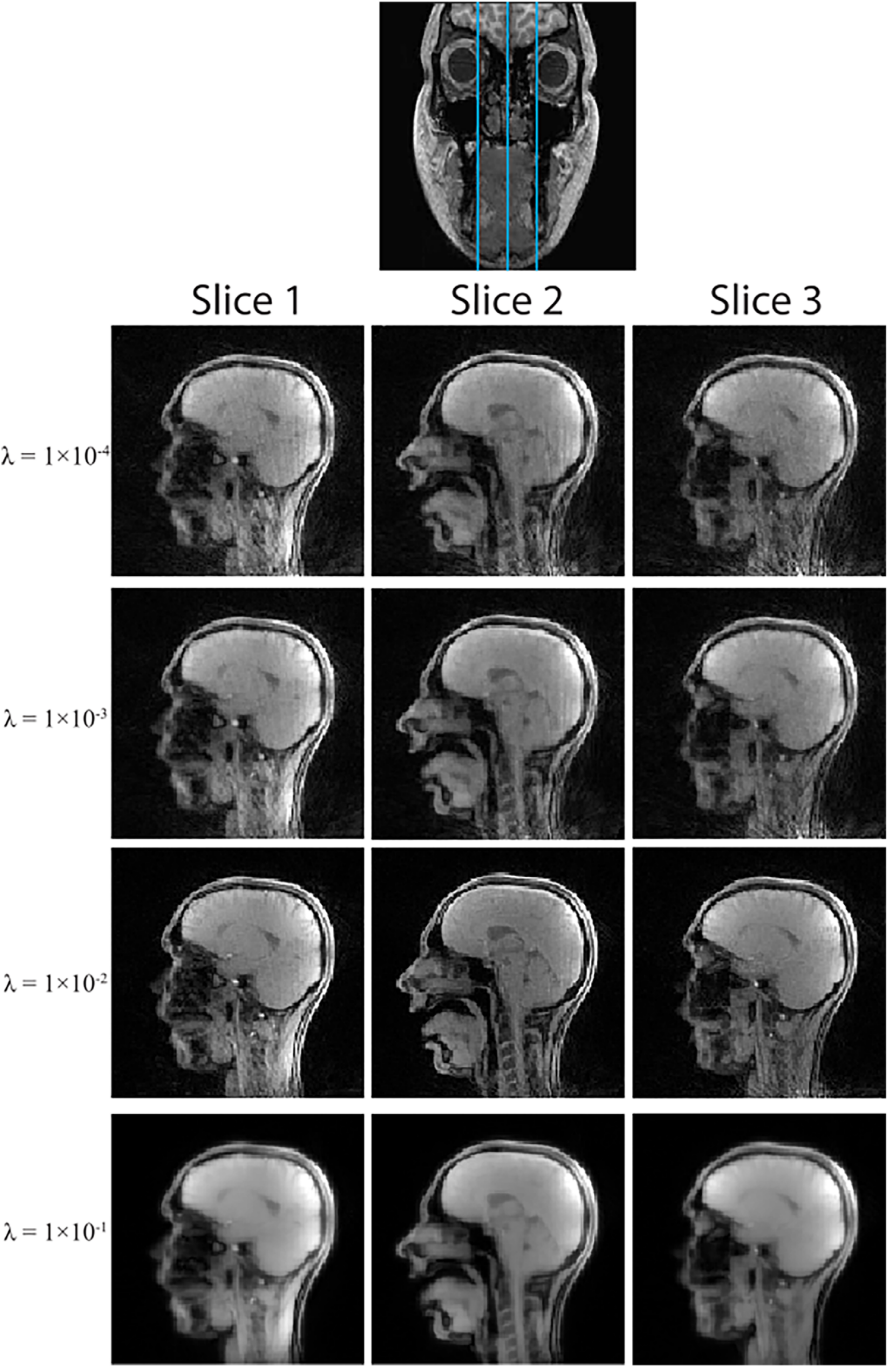
Results (showing all 25 spokes/frame, three slices, 12 mm apart; blue lines through coronal slice at the top of image show the approximate slice locations) from the coarse λ search. From this coarse search, it can be seen that the optimum λ value lies between the range of λ = 1 × 10^−2^ and λ = 1 × 10^−1^.

Results from the coarse search of λ values are shown in Figure 3, and results from the finer search are shown in Figure S5. Based on visual inspection of edge sharpness, perceived SNR, and temporal fidelity, a value of λ = $4\times 10^{-2}$ was chosen. This value was used throughout the rest of this work.

### 3.2 Comparison of sampling schemes and reconstruction methods

**FIGURE 4:**
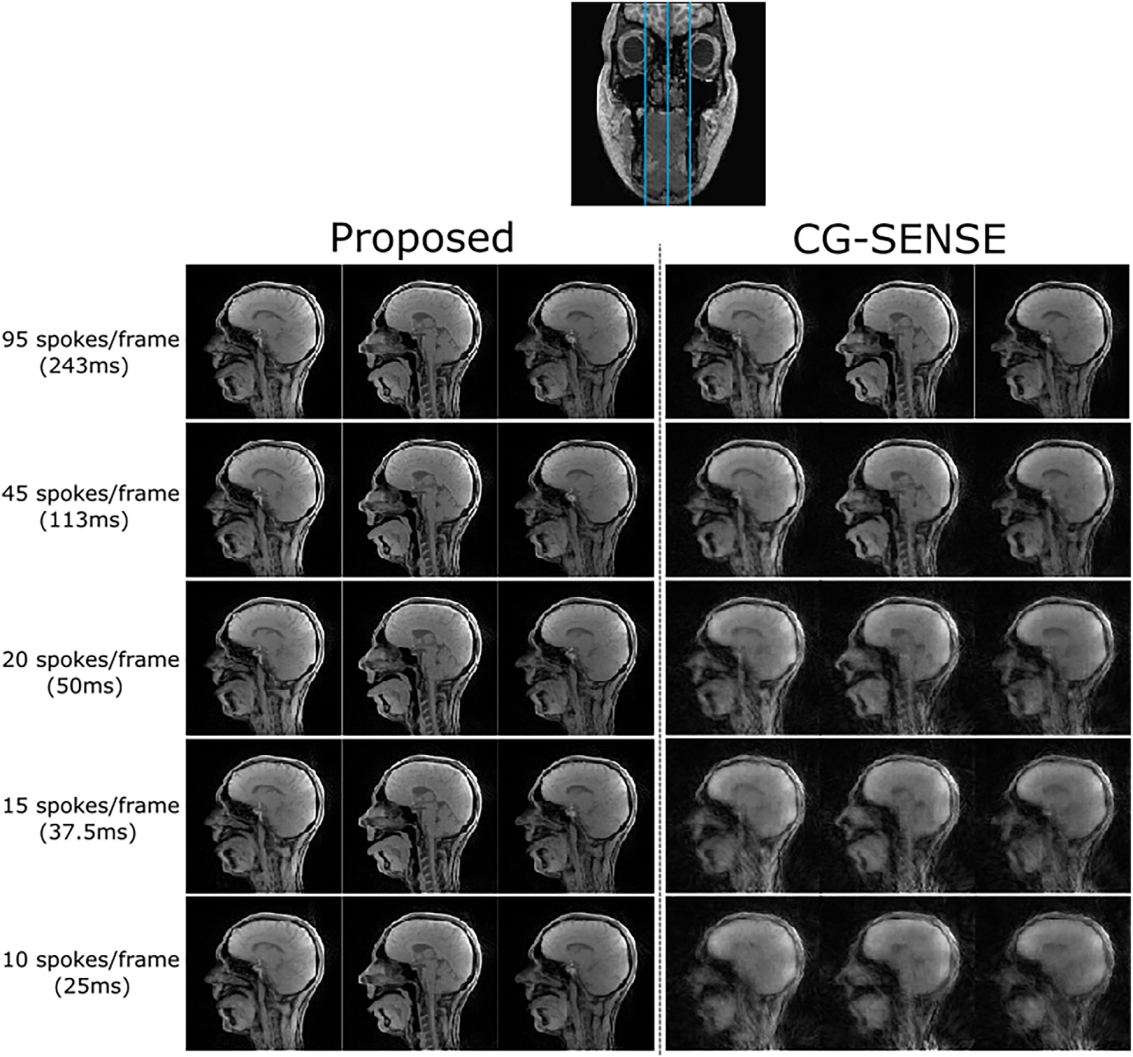
A comparison of two reconstruction algorithms (proposed and CG‐SENSE) at a variety of undersampling levels (from top to bottom: 95, 45, 20, 15, and 10 spokes/frame). The data used in this experiment is acquired using SMS GA sampling with a slice distance of 4.8 mm; the blue lines through the coronal slice at the top of the figure indicate the approximate positions of the three slices. The ADMM algorithm results in higher image quality at the highest levels of undersampling. ADMM, alternating direction method of multipliers; CG‐SENSE, conjugate gradient SENSE.

In vivo images reconstructed from data acquired using SMS GA sampling, at increasing levels of undersampling, are shown in Figure 4 (full rtMRI videos are shown in Video S3). This figure compares to the proposed reconstruction approach to the CG‐SENSE algorithm (15 iterations), originally used by Yutzy et al. for radial CAIPIRINHA SMS reconstruction.^9^ The undersampling experiment was repeated using the GA sampling scheme (Figure S6 and Video S4). In both sampling schemes, the proposed compressed sensing reconstruction algorithm results in improved image quality in terms of perceived SNR and structural details, particularly when low numbers of spokes are used.

In the rtMRI videos (see supplementary data), variations in image intensity between frames can be seen. These variations increase as the undersampling factor increases. This is likely due to destructive interference introduced by CAIPIRINHA modulation not being fully removed by the reconstruction algorithm. The intensity variations are more intense when GA sampling is used compared to SMS GA sampling.

Additional phantom results comparing both sampling schemes are described in Text S1 and Video S2. The use of the SMS GA sampling scheme shows a reduced amount of slice leakage compared to the GA sampling scheme. This is particularly noticeable in the central slice of the rtMRI phantom data.

**FIGURE 5:**
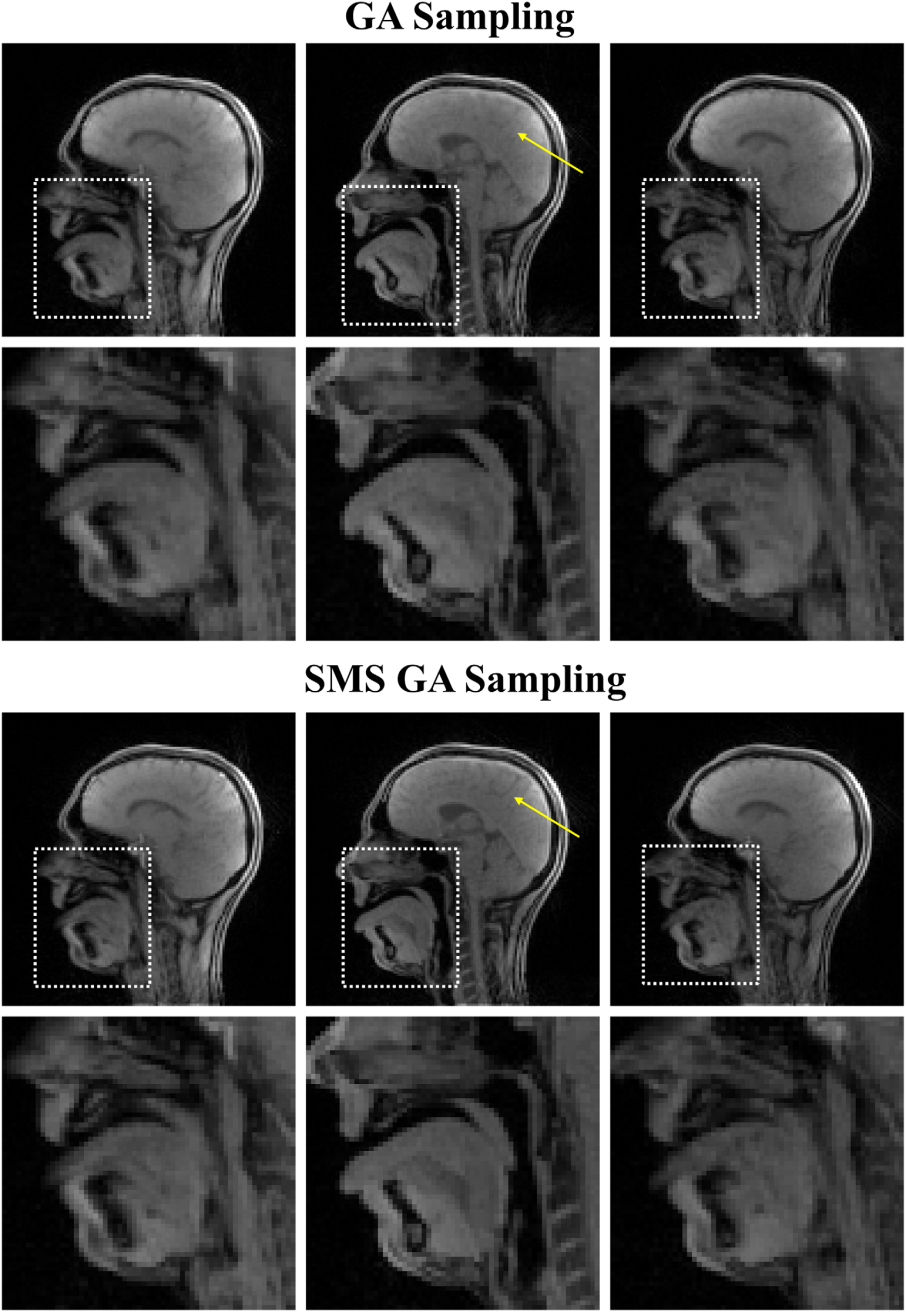
Comparison of a frame (15 spokes/frame, temporal resolution 37.5 ms) reconstructed using the proposed reconstruction pipeline from data acquired using GA sampling (top) and SMS GA sampling (bottom). Both the whole image and the oral cavity (region indicated by the white dotted box) are shown. Both frames appear visually very similar with the bottom frame having slightly more fine detail visible in the brain. The yellow arrow indicates an area with visible difference. In the SMS GA sampling scheme, the finer details of cortical folds appear sharper compared to the GA sampling scheme.

**FIGURE 6:**
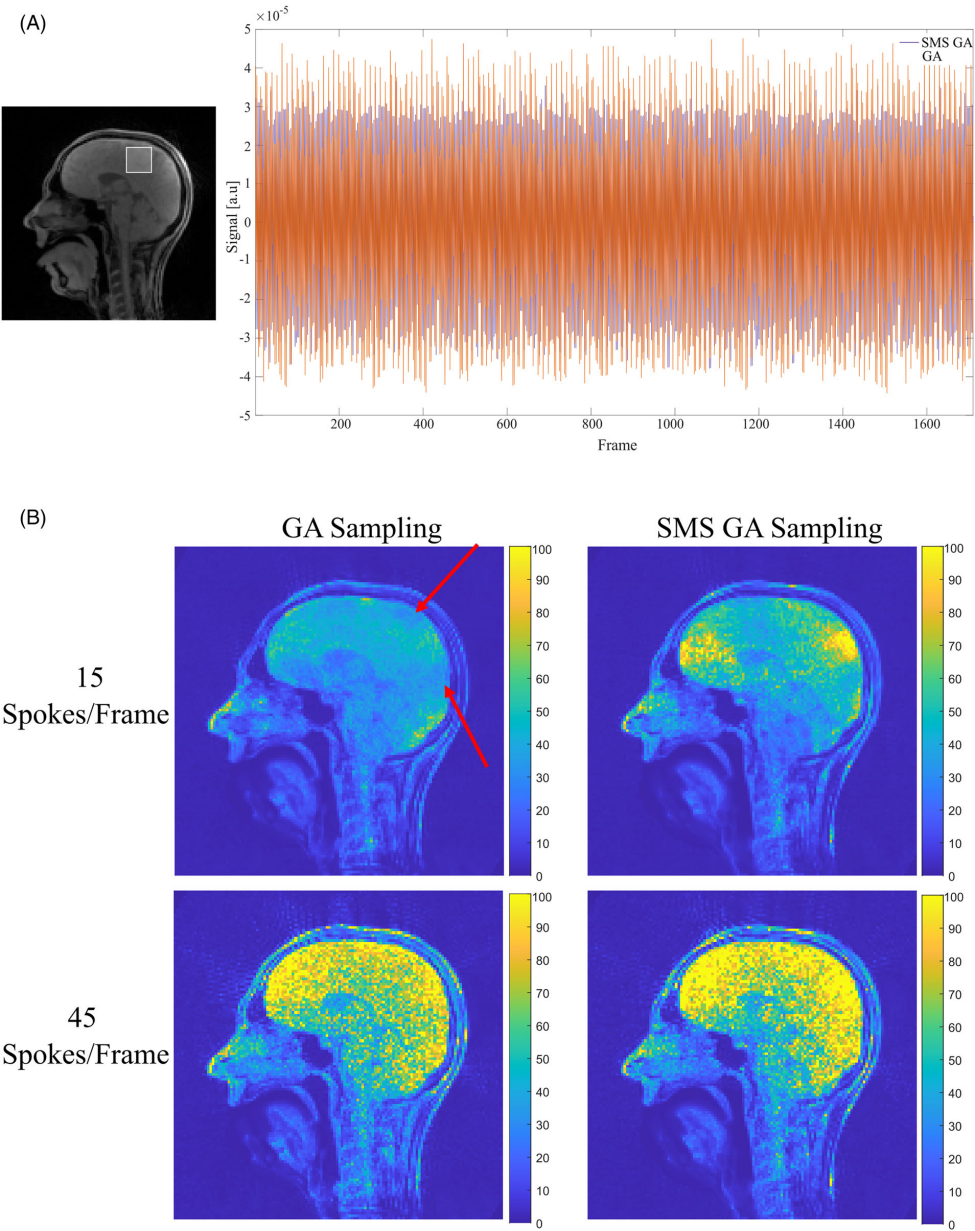
(A) Plot of the signal variations (normalized to a zero mean) in the ROI (delimited by the white box) for GA sampling (orange, RMS = 2.416 × 10^−5^) and SMS GA sampling (blue, RMS = 2.409 × 10^−5^) when 15 spokes/frame are used. The use of GA sampling results in larger fluctuations of signal intensity. (B) Top: tSNR of the central slice of three‐slice SMS rtMRI data reconstructed, using the proposed sampling scheme, with 15 spokes/frame (temporal resolution: 37.5 ms) acquired using the GA (left) and SMS GA (right) sampling schemes. Bottom: SNR of the central slice of three‐slice SMS rtMRI data reconstructed with 45 spokes/frame (temporal resolution: 123 ms) acquired using the GA (left) and SMS GA (right) sampling schemes. In both rows, the intensity is scaled to maximum of 100 to highlight differences between the two sampling schemes. The red arrows indicate areas of low tSNR in the 15 spokes/frame GA sampling scheme. ROI, region of interest; tSNR, temporal SNR.

A visual comparison between both sampling schemes (15 spokes/frame) is shown in Figure 5. The overall image quality in the two sampling schemes is qualitatively very similar; the SMS GA sampling scheme shows some finer anatomical structures, particularly in the brain. However, this comparison is only between a single frame and does not highlight the intensity variations that are visible in the rtMRI videos. Video S5 directly compares SMS GA and GA sampling at a range of temporal resolutions. In this video, the rtMRI videos acquired using GA sampling have an increased level of intensity variation (particularly at high levels of undersampling) compared to the equivalent video acquired using SMS GA sampling. To visualize these variations, two approaches are used. First, for both sampling schemes, the signal in a region of interest was calculated for 15 spokes/frame data and normalized to have a zero mean. This is shown in Figure 6 (top); the GA sampling scheme shows higher levels of signal variation compared to the SMS GA sampling scheme. Additionally, the temporal SNR (tSNR) of each pixel was calculated for 15 spokes/frame and 45 spokes/frame rtMRI data acquired using both sampling schemes.^28^ One expects static areas, such as the brain region, to have a high tSNR because the SD of intensity should be low. The results of tSNR calculations are shown in Figure 6 (bottom); at 15 spokes/frame, the SMS GA sampling scheme has an overall higher and more homogenous tSNR. In comparison, the tSNR images for GA sampling (at 15 spokes/frame) show regions of low tSNR. At 45 spokes/frame (where only minor intensity variations are visible in rtMRI videos for either sampling scheme), the tSNR is high for both sampling schemes.

### 3.3 Effect of slice gap

**FIGURE 7:**
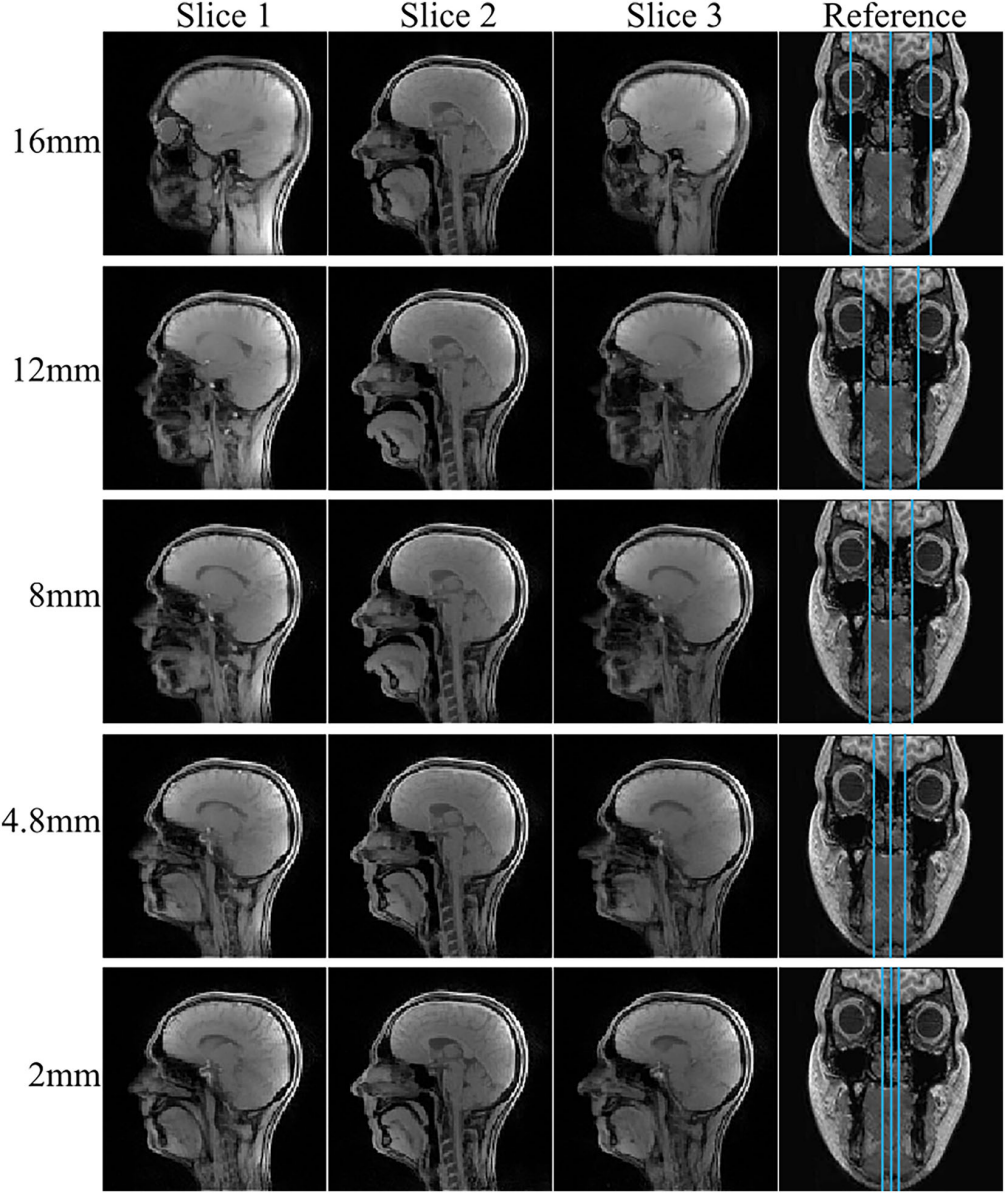
Images (three slices, 25 spokes/frame) reconstructed with the proposed reconstruction pipeline using data obtained with SMS GA sampling at a range of slice distances. From top to bottom 16 mm‐, 12 mm‐, 8 mm‐, 4.8 mm‐, and 2 mm‐slice distances. No artifacts due to slice leakage are visible.

In certain applications, such as imaging tongue movement, it is desirable to have a small distance between slices. In this work, we define slice distance as the gap between the closest edges of adjacent slices. Figure 7 (Video S5) compares SMS rtMRI images (acquired with the SMS GA trajectory at 15 spokes/frame and 8 mm‐slice thickness) across an array of slice distances: 16 mm, 12 mm, 8 mm, 4.8 mm, 2 mm. No visible artifacts that could compromise image quality were observed at this level of undersampling. Equivalent results for the GA sampling scheme are shown in Figure S7.

### 3.4 Computation time

Figure S7 compares the reconstruction time (for the entire 20s rtMRI video) of the proposed reconstruction method and CG‐SENSE at increasing levels of coil compression for 25 spokes/frame. The proposed reconstruction method has a significantly higher reconstruction time; a linear fit shows a per coil reconstruction time of 126.3 s using the proposed method compared to 12.03 s using CG‐SENSE. The effect of compression on image quality was measured using the RMS error (RMSE) metric, where the uncompressed image was used as the ground truth. The RMSE plot in Figure S7 shows that the error begins to rapidly rise when the number of coils falls below 13. However, RMSE is not an ideal metric for this application because it will be heavily weighted by the brain region (rather than the oral cavity), which occupies much of the FOV. Visual examples of image quality at increasing levels of compression are shown in Figure S8 and Video S6. The selected level of compression (16 virtual coils) does not result in an observable visual difference compared to the uncompressed reference image.

### 3.5 Speech and swallowing applications

**FIGURE 8:**
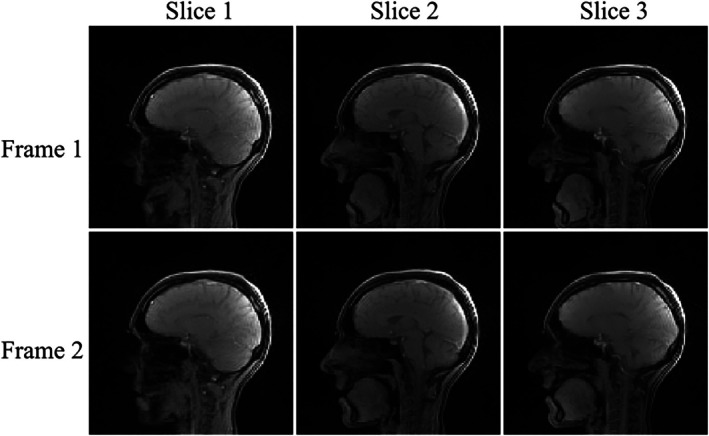
Two frames from an SMS rtMRI video of vowel formation. Each frame consists of three slices (12 mm‐slice distance). The images were reconstructed using the proposed reconstruction pipeline using the SMS GA sampling scheme (25 spokes/frame).

In addition to tongue mobility, rtMRI finds application in speech formation/linguistics research.^4^, ^29^, ^30^ Previous radial rtMRI single‐slice speech investigations, using specialized mouth/neck coil arrays, have been demonstrated at temporal resolutions of 33 ms.^30^ Here, with three slices imaged simultaneously, we record the pronunciation of English vowels with a temporal resolution of 62.5 ms (25 spokes/frame). This temporal resolution was selected to balance image quality (e.g., SNR and intensity variations) while being sufficient to capture that particular movement. In the rtMRI video (Figure 8 and Video S8), the tongue movements and lip seal required to pronounce each vowel can be clearly observed. The additional slices help to demonstrate the range of motions across the oral cavity, which occur during speech.

**FIGURE 9:**
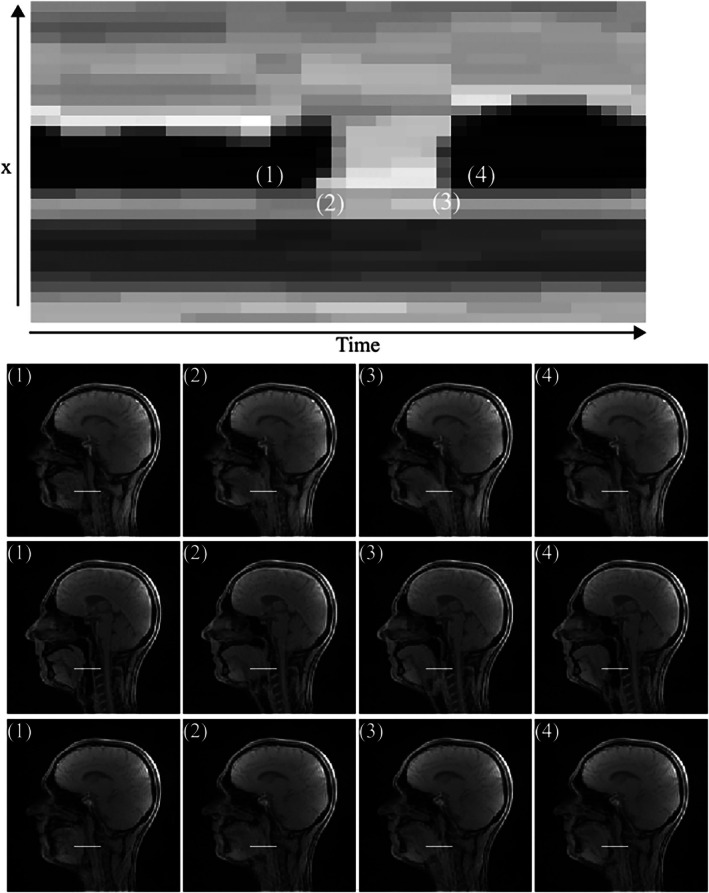
Top: x‐t plot (from central slice) of an empty swallow, the transportation of the bolus between (1) and (4) is visible. Bottom: Four frames from a three‐slice rtMRI (8 mm‐slice distance) acquisition. The white lines across the images indicate the position of the x‐t plot. The images were reconstructed using the proposed reconstruction pipeline and data was acquired using the SMS GA sampling scheme (25 spokes/frame).

Figure 9 shows the use of the proposed SMS rtMRI sequence (25 spokes/frame) to image the process of swallowing (Video S9). In this example, a volunteer performed an empty swallow. An x‐t plot (Figure 9, top) acquired from the central slice is used to visualize the oral transportation of the saliva.

## 4 DISCUSSION

We have demonstrated an SMS rtMRI sequence with a compressed sensing reconstruction pipeline. A FLASH sequence was chosen due its robustness to off‐resonance artifacts compared to balanced steady‐state free precession. This is particularly relevant in oral cavity imaging (at 3 T) due to the numerous air–tissue boundaries. The use of radial sampling, with a short readout duration (compared to spiral trajectories) improves robustness to off‐resonance effects. Examples of tongue movement imaging across three slices have been demonstrated at temporal resolutions of up to 25 ms at a spatial resolution of 2.2mm^2^. The use of CAIPIRINHA phase modulations and SMS GA sampling enabled slices distances of 2 mm to be achieved without visible degradation in image quality (however, quantitative evaluation of slice leakage has not been performed). Achieving a small slice separation can be important in some applications, such as recording tongue movements, because the structure/mobility of surgically reconstructed parts of the tongue may differ. The slice separation experiment results presented in section 3.3 used 15 spokes/frame, which provides sufficient temporal resolution for the applications demonstrated in this work. However, if further undersampling is desired, it is important to verify that the reconstruction algorithm can still effectively separate the slices at the increased undersampling level. To ensure this, the slice separation experiment should be repeated under the new sampling conditions.

In addition to imaging the movement of the tongue, examples of speech (specifically, imaging vowel formation) and swallowing imaging have been demonstrated. A temporal resolution of 62.5 ms was achieved, which satisfies the recommendations suggested by Lingala et al.^4^ The improved anatomical coverage, enabled by SMS acceleration, is beneficial in these applications because the anatomy required to enable these functions extends across the oral cavity. For example, in the swallowing rtMRI video (see Video S8), the additional slices highlight the large‐scale motion of the tongue, which is required to force the saliva from the front to the back of the oral cavity. Using SMS rtMRI in swallowing applications, for example, may be beneficial to study structural asymmetry (and the resulting change in function) present after reconstructive surgery/radiotherapy, which is known to affect bolus transportation.^31^

In the context of speech imaging, most useful information is contained in a midsagittal slice.^4^ However, the additional spatial information obtained using the proposed sequence helps demonstrate both the large‐scale motions (e.g., tongue movement) and the finer motions (e.g., lip seal) that enable speech. Additionally, SMS rtMRI could be beneficial in observing specific speech tasks in which the articulations occur away from the midsagittal slice.^32^

In this work, we have focused on three‐slice acceleration to demonstrate an initial proof of concept. Provisional results at higher (five‐slice) SMS acceleration are shown in Video S10 and Text S2. The increase in SMS acceleration results in increased intensity variations in both sampling schemes (compared to three‐slice SMS acceleration at the same temporal resolution). However, these initial results use a λ value optimized for three‐slice acceleration and thus may not be truly representative of the achievable image quality. In applications where a lower temporal resolution is acceptable, the number of spokes could be increased to allow for a higher level of SMS acceleration. The low‐flip angle used allows for this higher slice acceleration to be achieved.

A limitation of this work is the lack of quantitative comparison of image quality between GA sampling and SMS GA sampling. Further work is needed to robustly evaluate which sampling scheme should be used depending on the range of parameters being used. For example, the bootstrapped SNR method, which has previously been used in cardiac dynamic imaging applications, could be adapted for SMS rtMRI.^33^, ^34^

The regularization value was fixed at λ = $4\times 10^{-2}$ based on the search approach described in section 3.1. Our results show that this regularization parameter was suitable for a wide range of temporal resolutions and imaging applications. From the fine λ search figure (Figure S5), the regularization parameter selected has only minor differences (slightly increased edge sharpness and perceived SNR) compared to the coarse value of λ = 1 × 10^−2^. This may indicate that if some degradation in image quality is acceptable, only the coarse search is required. This would reduce the time needed to find a suitable regularization parameter, which in turn may be important when trying to further tune this methodology for different anatomical areas, applications, and sequence parameter combinations.

The proposed compressed sensing reconstruction pipeline was compared against CG‐SENSE reconstruction (with no regularization). Because the CG‐SENSE does not exploit any temporal redundancy, it is not surprising that it performs worse than the proposed compressed sensing reconstruction. Further work comparing compressed sensing to CG‐SENSE with temporal regularization (e.g., PCA/singular value decomposition regularization) is needed.

A disadvantage of the proposed approach is the approximately 10‐fold increase in reconstruction time compared to CG‐SENSE. Channel compression and GPU implementation of the NUFFT are currently used to reduce the computational burden. Further reductions in computation time could be achieved using more efficient implementations of the NUFFT.^35^ In addition, reconstruction time can be reduced and image quality improved using region‐specific channel compression techniques such as the ROVir compression method introduced by Kim et al.^36^ This has previously been demonstrated in SMS cardiac cine imaging.^37^

The current reconstruction pipeline exploits sparsity in the temporal dimension. The use of more complex regularization, such as PCA regularization, locally low rank or sparse and low‐rank techniques, may enable improved image quality at higher acceleration.^38^ This will further increase the computational burden required for reconstruction. Additionally, spiral sampling, which has improved sampling efficiency compared to radial sampling, will be investigated. SMS spiral sampling has shown promise in cardiac MRI applications such as cine and quantitative mapping.^11^, ^15^, ^39^, ^40^ However, in the context of rtMRI of the head/neck, additional correction techniques are typically required to reduce off‐resonance artifacts caused by multiple air–tissue and tissue–tissue boundaries.^41^ We have focused on using SMS acceleration; this is a multi‐slice technique rather than a fully volumetric acquisition. Comparing 3D non‐Cartesian/hybrid trajectories, such as stack‐of‐stars and koosh ball trajectories, to the proposed methodology is an area of future work.

## 5 CONCLUSION

This work has introduced a SMS rtMRI sequence and reconstruction pipeline capable of acquiring three slices simultaneously at a temporal resolution of up to 25 ms. We have demonstrated the sequence in a variety of head/neck imaging applications such as tongue mobility testing, speech, and monitoring (empty) swallowing. This may be useful in areas such as assessing function pre‐ and postsurgical intervention, and speech therapy. The proposed methodology could be applied to investigations of other body regions. For example, imaging of knee joint motion can be used to inform biomechanical models via real‐time moment arm calculations.^42^ The proposed sequence may also be beneficial in imaging hand/wrist movements, for example, in assessing and monitoring wrist instability.^43^

In addition to improving reconstruction at high acceleration levels, further work is required to optimize sequence parameters (e.g., the temporal resolution) depending on the application and anatomy of interest. It has previously been shown that dedicated mouth/neck coils can yield higher image quality. Importantly for clinical adoption, all results shown in this work have used a commonly available commercial head/neck coil (typically used for neuroimaging applications) rather than the dedicated mouth/neck coils used in previous speech rtMRI studies.^30^, ^44^

## FUNDING INFORMATION

i.w. received a PhD studentship from the School of Physics, Engineering and Technology, University of York.

We acknowledge partial funding of our work by a grant from the British Association of Oral and Maxillofacial Surgeons Endowments Committee (BAOMS).

## Supporting information


**Data S1.** Word document containing all supplementary information and supplementary figures mentioned in the main text
**Figure S1.** Making of the components of the phantom. A: Actuation method, shown on early prototype with fixed‐base tongue only. Insets show the embedded steering tip and tongue geometry. B: Backplate view of full phantom assembly with mandible and cheeks. Tongue i. Making of the components of the phantom. A: Actuation method, shown on early prototype with fixed‐base tongue only. Insets show the embedded steering tip and tongue geometry. B: Backplate view of full phantom assembly with mandible and cheeks. Tongue is now on a separate backplate (1), hinged from an acrylic rod (2) along with the mandible (3). Clamping bracket (4) secures the phantom in the head coil. C: Front view of full assembly. Mandible is actuated by the lower control cable that passes round a pulley on the backplate (5). Cheeks are suspended from the palate plate (6) and join underneath the mandible to form the floor of the mouth. D: Multipart mould for silicone cheeks casting; inset shows resulting silicone.
**Figure S2.** A comparison of GA sampling and SMS GA sampling, using a dynamic phantom, at increasing levels of undersampling. The yellow arrow indicates an region of increased noise when GA sampling is used. All images have been normalized to have the same maximum intensity.
**Figure S3.** Photographs of the phantom's mandible with small and medium sized surgical plates attached.
**Figure S4.** Sagittal and axial rtMRI images of the dynamic phantom without metal plates (top) and with metal plates (bottom). The white arrows indicate the approximate position of the surgical plates.
**Figure S5.** Results from a fine λ search (only central slice shown) with λ values increasing from λ = 1×10^−2^ to λ = 1×10^−1^ in steps of 1×10^−2^. Past λ = 5×10^−2^ blurring is visible.
**Figure S6.** A comparison of two reconstruction algorithms (proposed and CG‐SENSE) at a variety of undersampling levels (from top to bottom 95 45, 20, 15 and 10 spokes/frame). The data used in this experiment is acquired using GA sampling with a slice distance of 4.8mm, the blue lines through the coronal slice at the top of the figure indicate the approximate positions of the three slices. The proposed reconstruction algorithm results in higher image quality at the highest levels of undersampling.
**Figure S7.** Images (3 slices, 25 spokes/frame) reconstructed using data obtained with GA sampling at a range of slice distances. From top to bottom: 16mm, 12mm, 8mm, 4.8mm and 2mm slice distances. No artefacts due to slice leakage are visible.
**Figure S8.** The effect of coil compression on SMS rtMRI data (3 slices, 2mm slice distance) at varying levels of coil compression. The top left image (the central slice of the 3 slices) is reconstructed using all 64 coils. The compression level is then increased, with the number in the top left corner indicating the number of virtual coils used. The left images are a frame reconstructed from the compressed data and the right image is the absolute difference between the frame reconstructed using the compressed data and the reference image (using all coils).
**Figure S9.** Plot of reconstruction time (in seconds) at a range of coil compression levels for both reconstruction techniques. The proposed methods reconstruction time (126.3s per coil) (black) is substantially higher than the CG‐SENSE reconstruction time (red, 12.03s per coil). The root‐mean squared error between the compressed and uncompressed data is also plotted (green). An undersampling level of 25 spokes/frame is used for the data shown in this plot.
**Figure S10.** A frame from five slice rtMRI videos acquird with SMS GA sampling (top) and GA sampling (bottom) with a slice distance of 8mm and 45 spokes/frame. The coronal image (bottom) indicates the approximate slice positions.
**Figure S11.** Comparison of three frames (15 spokes/frame) acquired using the GA sampling scheme (top) and SMS GA sampling scheme (bottom). The central slice from a 5‐slice acquisition is shown. Blurring and artefacts are present in both sampling schemes. An example of one of these artefacts is indicated by the yellow arrow.
**Figure S12.** Comparison of three frames (25 spokes/frame) acquired using the GA sampling scheme (top) and SMS GA sampling scheme (bottom). The central slice from a 5‐slice acquisition is shown.
**Figure S13.** Comparison of three frames (25 spokes/frame) acquired using the GA sampling scheme (top) and SMS GA sampling scheme (bottom). The central slice from a 5‐slice acquisition is shown.
**Figure S14.** tSNR comparison of GA sampling and SMS GA sampling at different levels of undersampling. This shows the degradation in tSNR, for both sampling schemes, as the number of spokes/frame is decreased.

**Video S1.** Video showing how the phantom can be manipulated through the pulling of nylon wires attached to anchoring points in the phantom.

**Video S2.** A comparison of GA sampling (top) and SMS GA sampling (bottom) at 15, 25 and 45 spokes/frame using the dynamic phantom.

**Video S3.** Comparison of the proposed compressed sensing reconstruction pipeline and CG‐SENSE, at a range of temporal resolutions, for data acquired using the SMS GA sampling scheme.

**Video S4.** Comparison of the proposed compressed sensing reconstruction pipeline and CG‐SENSE, at a range of temporal resolutions, for data acquired using the GA sampling scheme.

**Video S5.** Comparison of the SMS GA and GA sampling, reconstructed using the proposed reconstruction pipeline, at a range of temporal resolutions.

**Video S6.** SMS rtMRI videos at a variety of slice distances.

**Video S7.** The effect of coil compression on image quality for SMS rtMRI data (25 spokes/frame).

**Video S8.** SMS rtMRI video (25 spokes/frame) of a volunteer pronouncing all English vowels.

**Video S9.** SMS rtMRI video (25 spokes/frame) of a volunteer swallowing saliva.

**Video S10.** Example of a five slice SMS rtMRI video of tongue movement at a variety of temporal resolutions.

## Data Availability

Reconstruction code and some k‐space data will be available upon publication at: https://github.com/iw596/SMSRealTimeMRI. The code has been developed and tested on a Windows 11 machine with a Nvidia 4070 GPU (Cuda Version V12.2.140) and MatLab version R2023b. All files required to reproduce the phantom are available on the following Github repository: https://github.com/iw596/Dynamic‐Phantom.git.
