# Supplementary material for: Extending real‐time MRI of the oral cavity using simultaneous multislice and compressed sensing
Source: Magn Reson Med. 2025 Sep 15;95(2):881–96. doi: 10.1002/mrm.70085 (PMC12681314; doi:10.1002/mrm.70085)
Supplement: Supplementary file 1 — Data S1. Word document containing all supplementary information and supplementary figures mentioned in the main text Figure S1. Making of the components of the phantom. A: Actuation method, shown on early prototype with fixed‐base tongue only. Insets show the embedded steering tip and tongue geometry. B: Backplate view of full phantom assembly with mandible and cheeks. Tongue i. Making of the components of the phantom. A: Actuation method, shown on early prototype with fixed‐base tongue only. Insets show the embedded steering tip and tongue geometry. B: Backplate view of full phantom assembly with mandible and cheeks. Tongue is now on a separate backplate (1), hinged from an acrylic rod (2) along with the mandible (3). Clamping bracket (4) secures the phantom in the head coil. C: Front view of full assembly. Mandible is actuated by the lower control cable that passes round a pulley on the backplate (5). Cheeks are suspended from the palate plate (6) and join underneath the mandible to form the floor of the mouth. D: Multipart mould for silicone cheeks casting; inset shows resulting silicone. Figure S2. A comparison of GA sampling and SMS GA sampling, using a dynamic phantom, at increasing levels of undersampling. The yellow arrow indicates an region of increased noise when GA sampling is used. All images have been normalized to have the same maximum intensity. Figure S3. Photographs of the phantom's mandible with small and medium sized surgical plates attached. Figure S4. Sagittal and axial rtMRI images of the dynamic phantom without metal plates (top) and with metal plates (bottom). The white arrows indicate the approximate position of the surgical plates. Figure S5. Results from a fine λ search (only central slice shown) with λ values increasing from λ = 1×10^−2^ to λ = 1×10^−1^ in steps of 1×10^−2^. Past λ = 5×10^−2^ blurring is visible. Figure S6. A comparison of two reconstruction algorithms (proposed and CG‐SENSE) at a variety of undersampling levels (from top to bott [file MRM-95-881-s001.docx]

**Supplementary Text 1 Dynamic Phantom**

Simple phantoms cannot reproduce the rich and dense structural details and functions of certain parts of the human anatomy. Furthermore, static phantoms are of limited use in the testing of dynamic imaging techniques such as real-time MRI. These shortcomings can be addressed by using a more anatomically realistic phantom with biomimetic structure and movements. This can provide an intermediate stage in the development of an MRI methodology allowing it to be evaluated to a more advanced level before human subjects are required. The construction of the phantom is depicted in Figure S1. The acrylic frame of the phantom comprises a front and back plate joined by an upper palate plate and two lower struts. Nylon fasteners, rather than glue, are used to join the acrylic plates and struts to enable easy disassembly and storage.


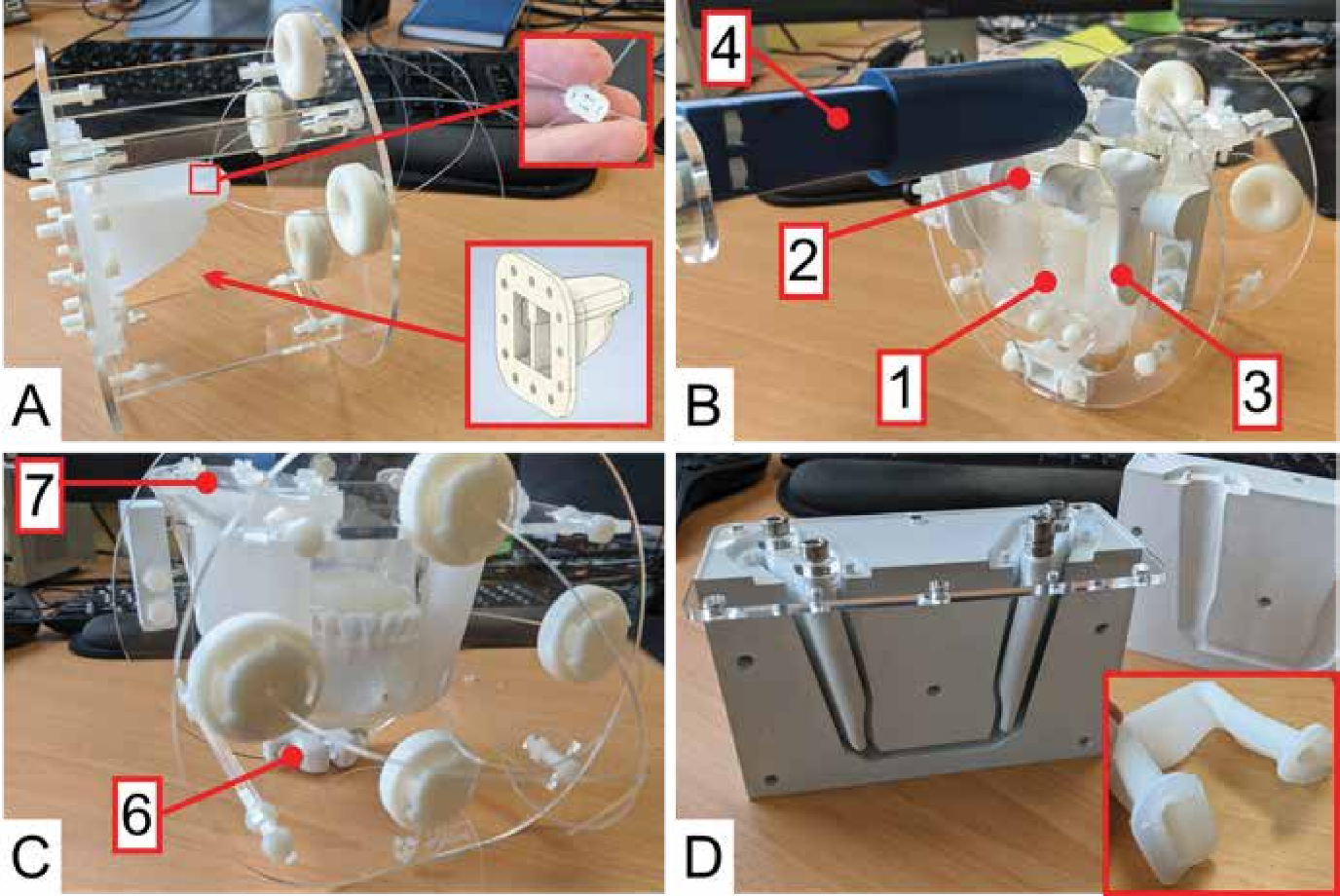


**Supplementary Figure 1**: Making of the components of the phantom. A: Actuation method, shown on early prototype with fixed-base tongue only. Insets show the embedded steering tip and tongue geometry. B: Backplate view of full phantom assembly with mandible and cheeks. Tongue i. Making of the components of the phantom. A: Actuation method, shown on early prototype with fixed-base tongue only. Insets show the embedded steering tip and tongue geometry. B: Backplate view of full phantom assembly with mandible and cheeks. Tongue is now on a separate backplate (1), hinged from an acrylic rod (2) along with the mandible (3). Clamping bracket (4) secures the phantom in the head coil. C: Front view of full assembly. Mandible is actuated by the lower control cable that passes round a pulley on the backplate (5). Cheeks are suspended from the palate plate (6) and join underneath the mandible to form the floor of the mouth. D: Multipart mould for silicone cheeks casting; inset shows resulting silicone**.**

All structural elements of the phantom are confined to a compact cylindrical volume, so that it may be used in a 30cm horizontal magnet bore, but also easily adapted for use in the head coils of larger scanners. In our setup, a 3D printed bracket enables it to be clamped securely to a rear slot in the head coil.

The tongue has two cavities for filling with agar gel, mounted and sealed by clamping its flanged base between two acrylic plates (having two separate compartments of the tongue mimics the tongue’s anatomy and a common post-surgical situation following partial resection and reconstruction of the tongue). Nylon cables tied to a plastic steering point are embedded in the tongue tip during the liquid-silicone casting process and pass through tapered 3D printed bushings mounted on the front plate. Puppet-like pulling of these cables thereby enables simple lateral movement in two axes, and the tongue can be stuck out by pulling multiple cables simultaneously. The cheeks each have a cavity for filling with agar gel, flanges like the tongue, and pass up and through holes in the palate plate to be secured and sealed by sandwiching the flanges between acrylic clamping plates and the topside of the palate. Clamping the flanges between acrylic plates prevents leakage from the agar gel-filled phantom cavities.

To incorporate mandibular motion, the tongue backplate and the mandible are suspended from an acrylic rod on the main backplate, allowing both parts to swing back around its axis. The lower control cable is attached to the chin and exits through the front plate via a pulley on the backplate, directing its pulling force along the correct line of action for opening the mouth. An opposite force is required to return it to the closed position, which is provided by the floor of the mouth upon which the mandible rests. This is integrated with the cheeks in a single silicone casting, such that the cheeks stretch when the mandible pivots downwards and thereby provide an elastic restoring force to pull the chin back up again.

The silicone parts are cast in 3D printed moulds with laser cut lids that allow holes for air to escape as they fill. Cavities for filling with agar gel are created by attaching 3D printed inserts to the lid, which project into the casting volume and hence leave behind voids when they are removed. Liquid silicone is injected upwards into a hole in the mould base to avoid trapping air bubbles, which is then plugged while the silicone cures. For the cast silicone to be removable, it must neither enclose, nor be enclosed by, plastic in the direction of removal. For this reason, the moulds have an open-ended design, and consist of multiple segments clamped together with bolts, such that the mould can be disassembled to release the casting.

The tongue, bilateral floor of the mouth and cheeks (for the 3D printing data sets see supplementary materials) were constructed from moulded silicone ‘skins’ (Smooth-On Ecoflex 00-20 FAST) and filled with agar hydrocolloid (Biozoon, Germany; concentration of 2g per 100ml of water). The mandible was derived from a 3D scanned STL mesh model of a human mandible (donated, from high-resolution CT scans), processed with Autodesk Meshmixer to form a printable hollow solid, and 3D printed on a Stratasys F170 using Stratasys acrylonitrile styrene acrylate (ASA) plastic (for the 3D printing data set see supplementary materials) to represent the cortical bone. Hollow spaces inside the mandibular structure were filled with plaster (West Design Products, UK) to represent cancellous bone in the mandible. The phantom frame was constructed using laser cut 3mm acrylic sheet (Hindleys, UK). M5 and M3 nylon fasteners were used to attach the different parts of the phantom to the frame. Nylon strings were attached to the tongue and jaw to provide a mechanism for moving them. The associated hinge and pivot parts were created from 6mm solid acrylic rod and 3D printed ASA plastic. All files required to reproduce the phantom are available on the following Github repository:

<https://github.com/iw596/Dynamic-Phantom.git>

The dynamic phantom was used to test the real-time MRI sequence (rtMRI). Two SMS rtMRI datasets were acquired using the GA and SMS GA sampling schemes. Three slices, covering the tongue and both cheeks, were acquired simultaneously. All data was reconstructed using the proposed compressed sensing reconstruction algorithm. Figure S2 compares the effect of undersampling when using the GA sampling and SMS GA sampling. Compared to GA sampling, the use of SMS GA sampling results in an increase in notice, particularly in the central slice. The regularization value used for reconstruction was the same as that used for in-vivo reconstruction. This value is not optimized for the dynamic phantom. Thus, the phantom results may not truly the reflect the best possible image quality.

*
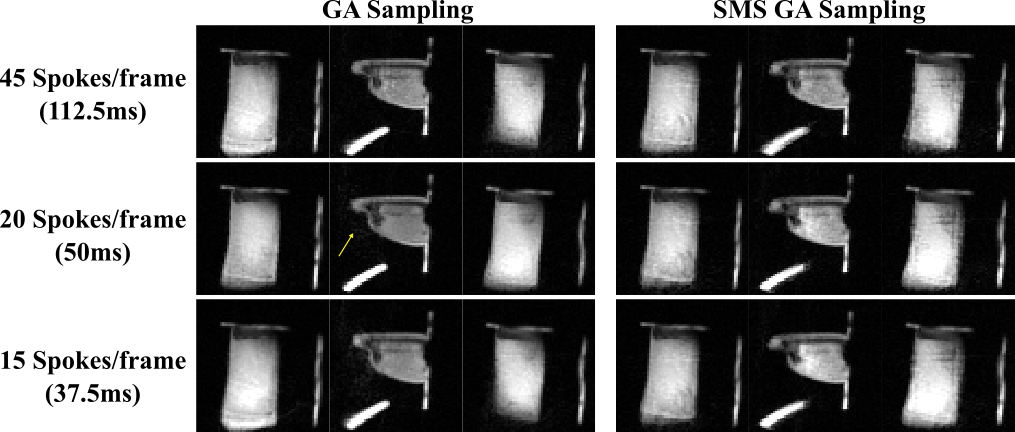
*

**Supplementary Figure 2**: A comparison of GA sampling and SMS GA sampling, using a dynamic phantom, at increasing levels of undersampling. The yellow arrow indicates an region of increased noise when GA sampling is used. All images have been normalized to have the same maximum intensity.

In addition to testing rtMRI sequences, the dynamic phantom enables investigations that are not typically possible with healthy volunteers. For example, exploring the effect of surgical plates on image quality. A demonstration of attaching surgical plates to the phantom is shown in Figure S3. From Figure S4, which compares the phantom with /without metal plates, it can be seen that the surgical plates do not affect the image quality. These results are not intended to be a rigorous study of off-resonance effects caused by the presence of surgical reconstruction plates but are intended to demonstrate the potential of a dynamic phantom for investigating the effect of reconstruction plates on image quality. Robustly investigating these effects in a controlled manner would be very difficult to achieve in patients or healthy volunteers.

**
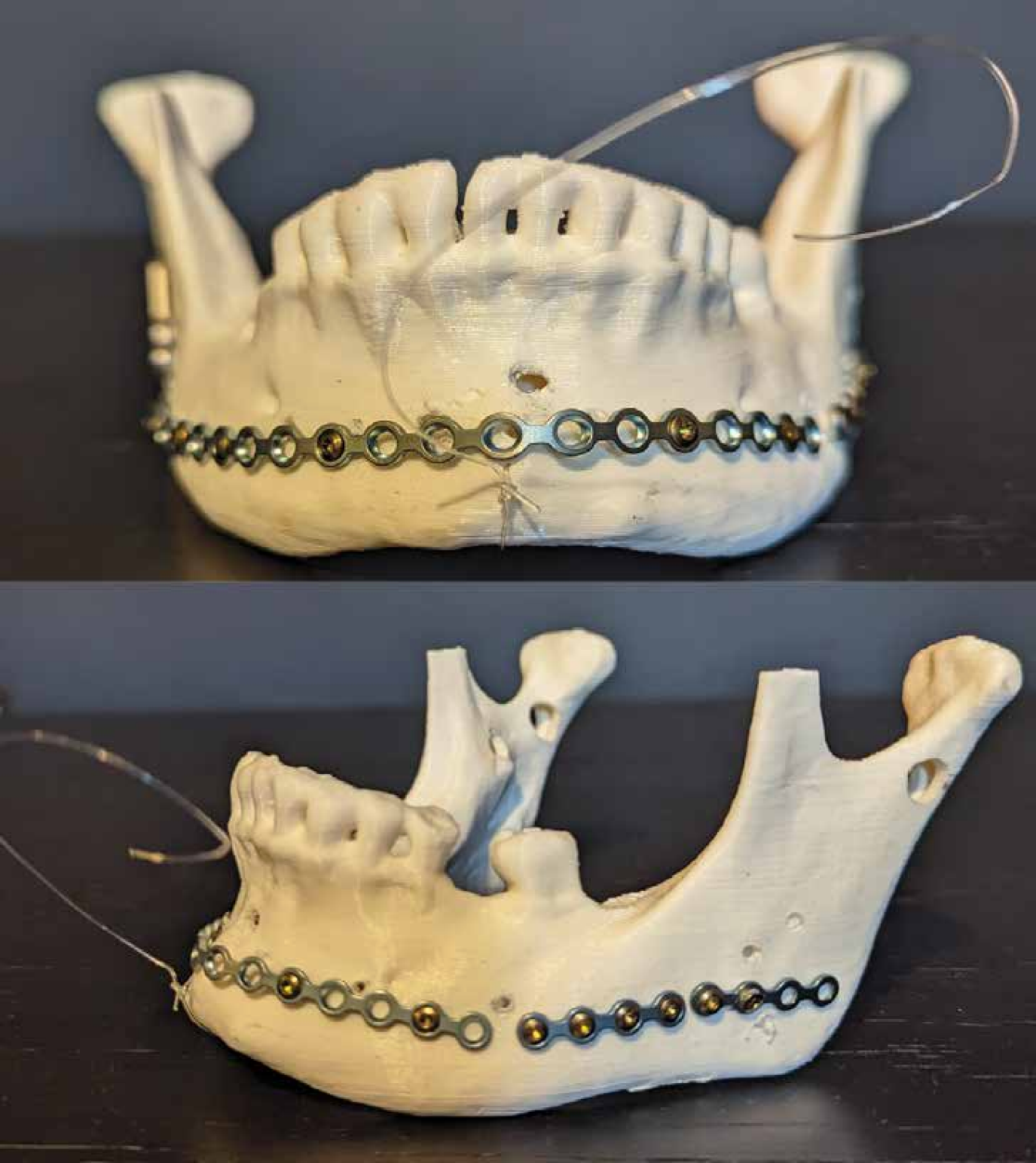
**

**Supplementary Figure 3:** Photographs of the phantom’s mandible with small and medium sized surgical plates attached.

**
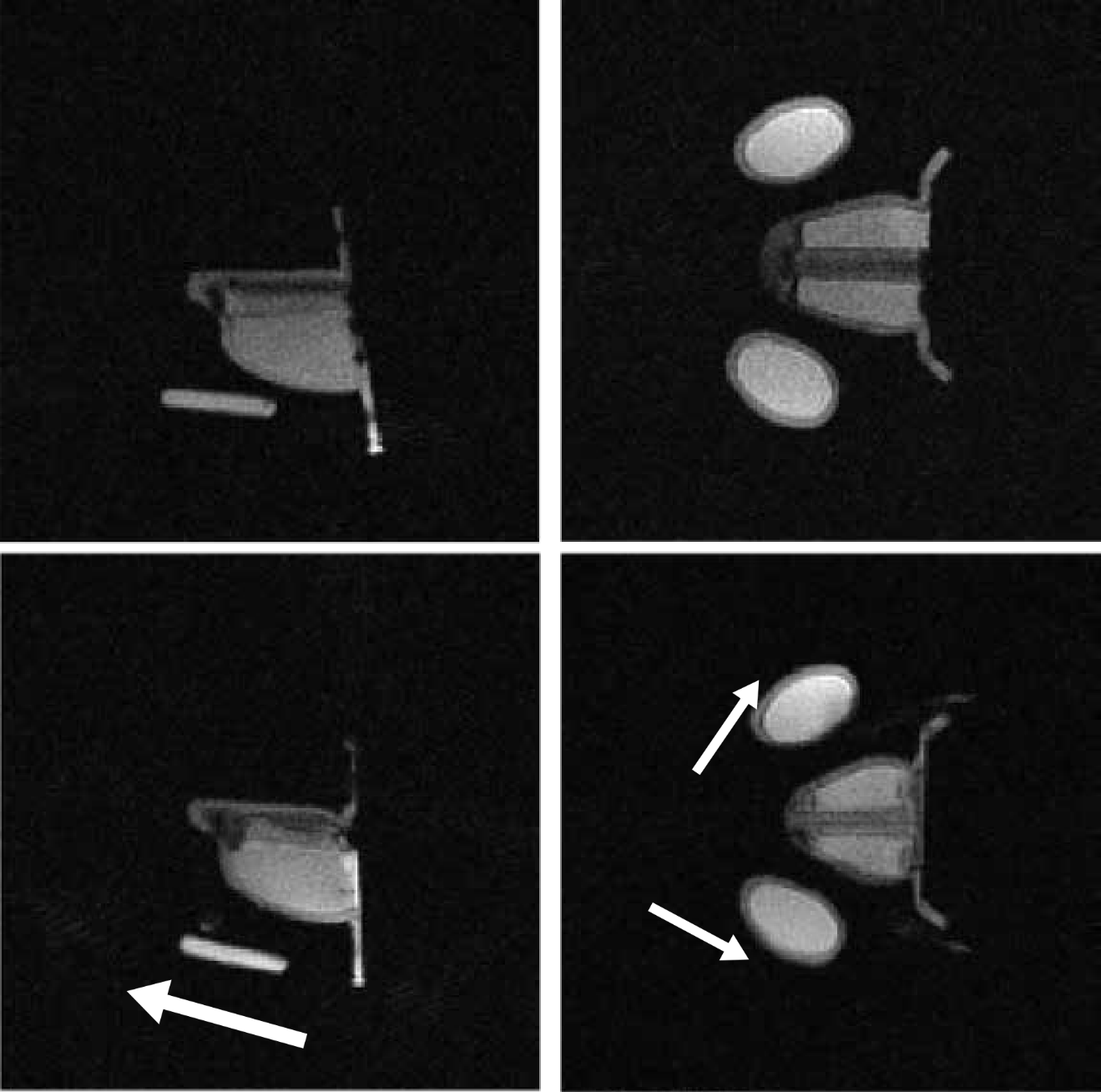
**

**Supplementary Figure 4:** Sagittal and axial rtMRI images of the dynamic phantom without metal plates (top) and with metal plates (bottom). The white arrows indicate the approximate position of the surgical plates.

**Fine regularization parameter search**


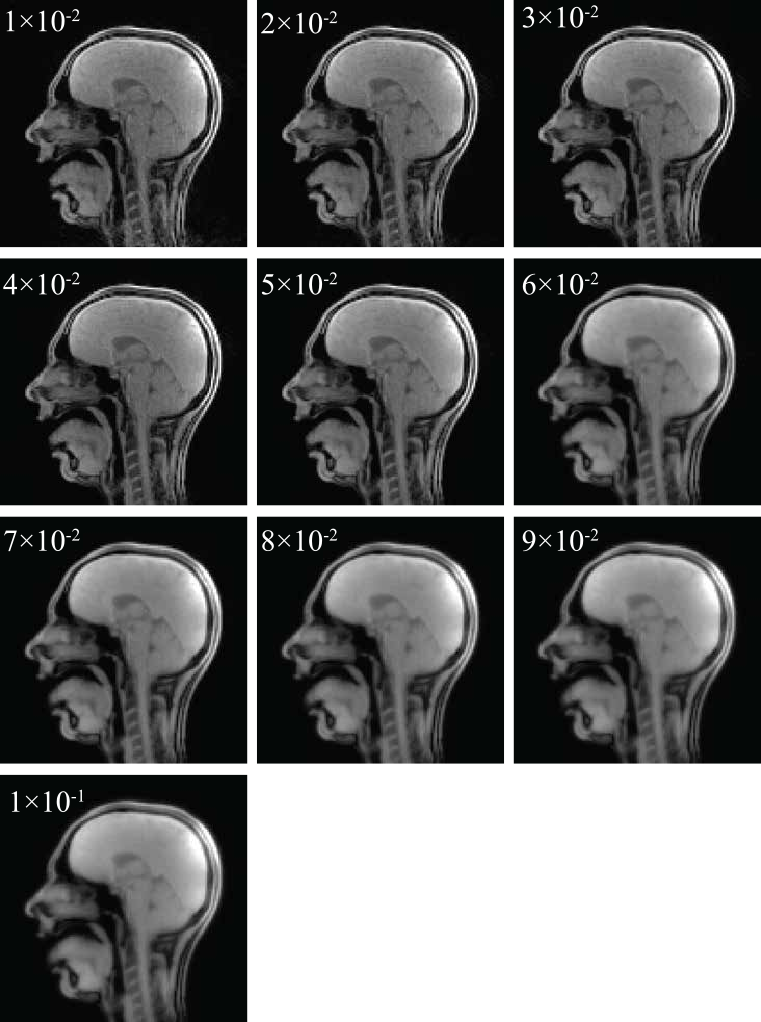


**Supplementary Figure 5**: Results from a fine λ search (only central slice shown) with λ values increasing from λ = 1×10^−2^ to λ = 1×10^−1^ in steps of 1×10^−2^. Past λ =5×10^−2^ blurring is visible.

**GA sampling results**


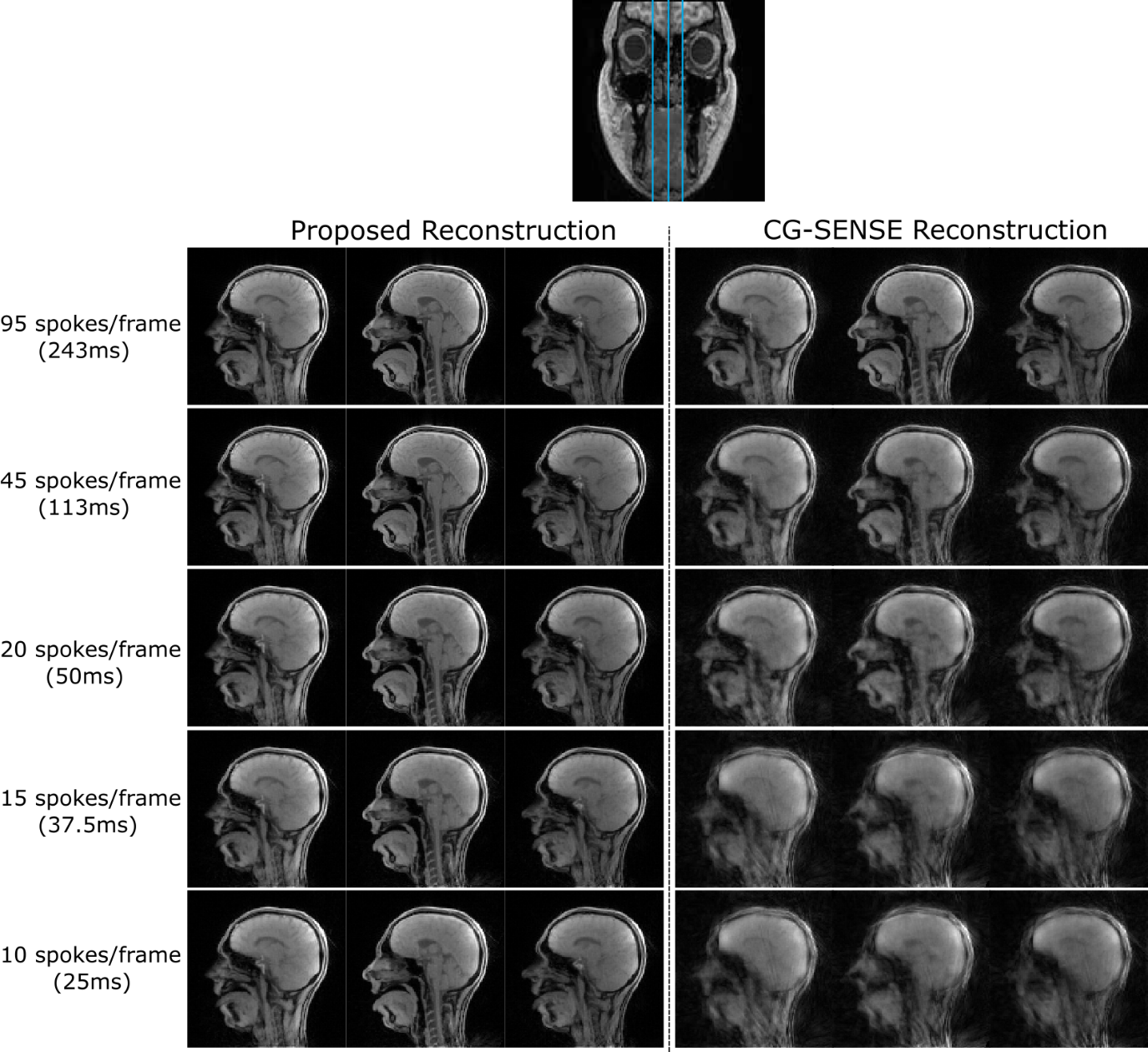


**Supplementary Figure 6**: a comparison of two reconstruction algorithms (proposed and CG-SENSE) at a variety of undersampling levels (from top to bottom 95 45, 20, 15 and 10 spokes/frame). The data used in this experiment is acquired using GA sampling with a slice distance of 4.8mm, the blue lines through the coronal slice at the top of the figure indicate the approximate positions of the three slices. The proposed reconstruction algorithm results in higher image quality at the highest levels of undersampling.

**GA Sampling Slice Distance Results**

**
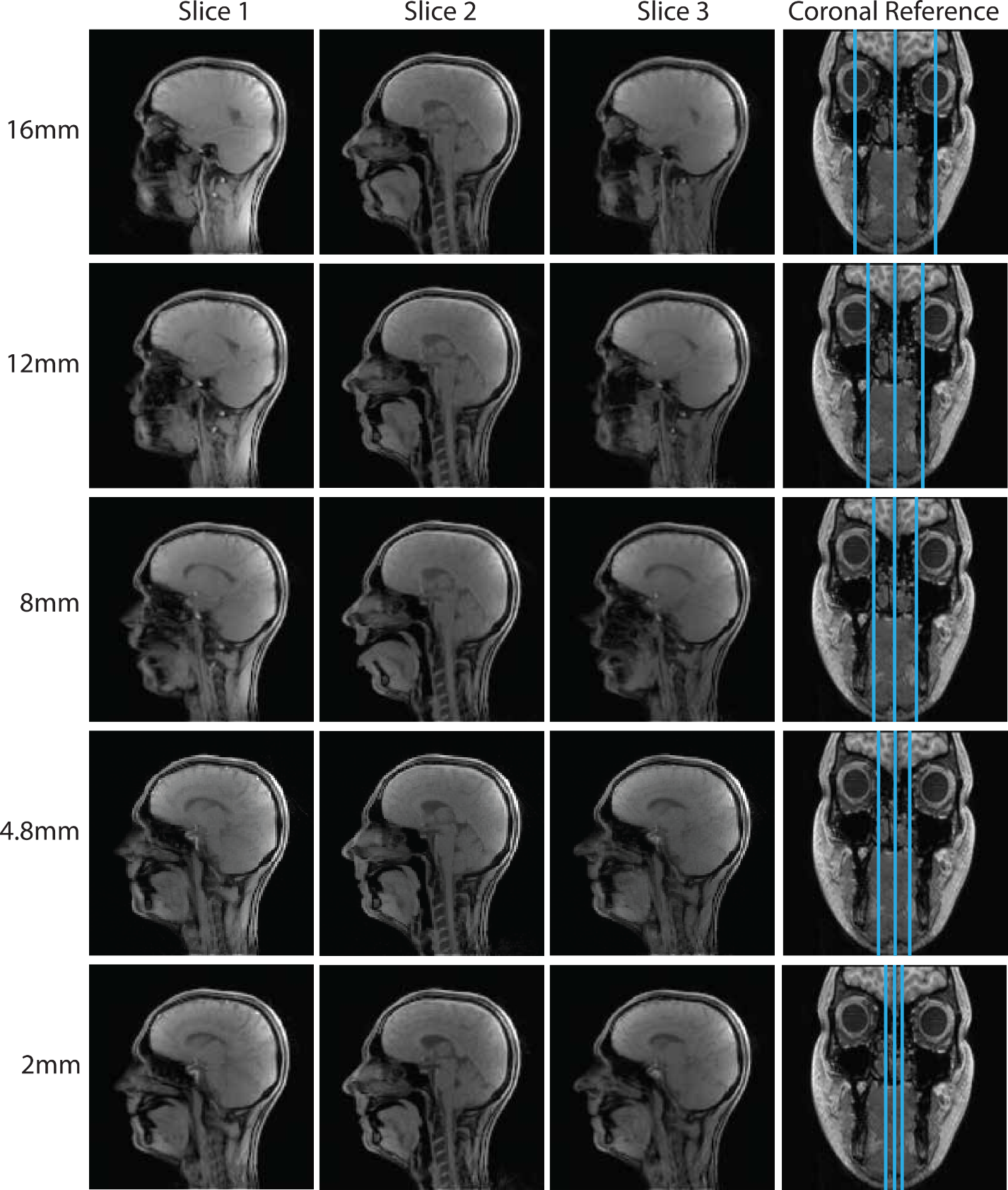
**

**Supplementary Figure 7:** Images (3 slices, 25 spokes/frame) reconstructed using data obtained with GA sampling at a range of slice distances. From top to bottom: 16mm, 12mm, 8mm, 4.8mm and 2mm slice distances. No artefacts due to slice leakage are visible.

**Coil Compression Image Quality Resutls**

**
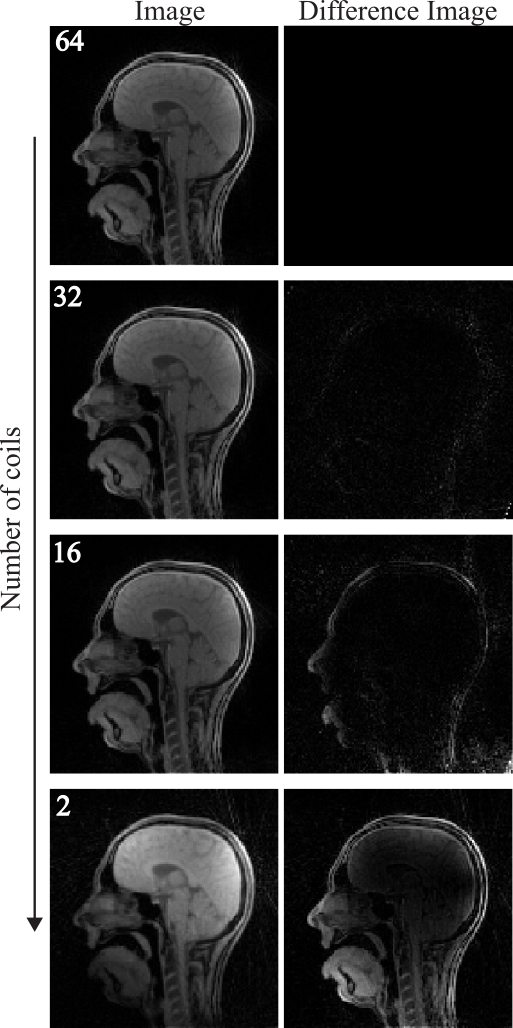
**

**Supplementary Figure 8:** The effect of coil compression on SMS rtMRI data (3 slices, 2mm slice distance) at varying levels of coil compression. The top left image (the central slice of the 3 slices) is reconstructed using all 64 coils. The compression level is then increased, with the number in the top left corner indicating the number of virtual coils used. The left images are a frame reconstructed from the compressed data and the right image is the absolute difference between the frame reconstructed using the compressed data and the reference image (using all coils).

**
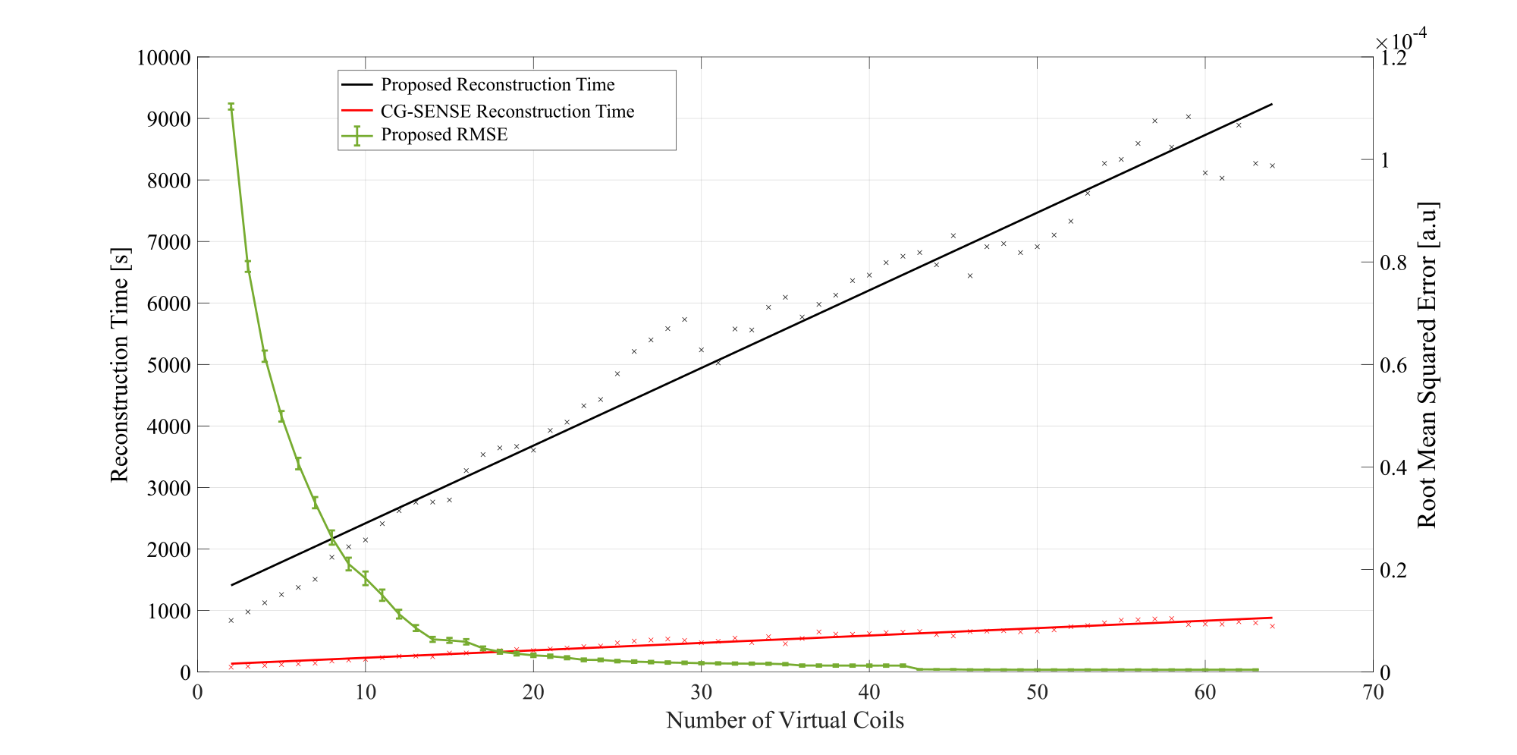
**

**Supplementary Figure 9:** Plot of reconstruction time (in seconds) at a range of coil compression levels for both reconstruction techniques. The proposed methods reconstruction time (126.3s per coil) (black) is substantially higher than the CG-SENSE reconstruction time (red, 12.03s per coil). The root-mean squared error between the compressed and uncompressed data is also plotted (green). An undersampling level of 25 spokes/frame is used for the data shown in this plot.

**Supplementary Text 2: Provisional Five Slice Acceleration Results**

To see the effect of data undersampling at this higher level of SMS acceleration, the undersampling experiment shown in Section 3.1 was repeated for the five slice rtMRI data (using both sampling schemes). All images shown in this section were reconstructed using the proposed reconstruction pipeline. The regularization value was identical to that of the three-slice images shown in the main text. Therefore, further improvement in image quality could likely be achieved using a regularization value tailored for five-slice SMS acquisition. Figure S10 shows the anatomical coverage achieved when five slices are acquired (with an 8mm slice distance).


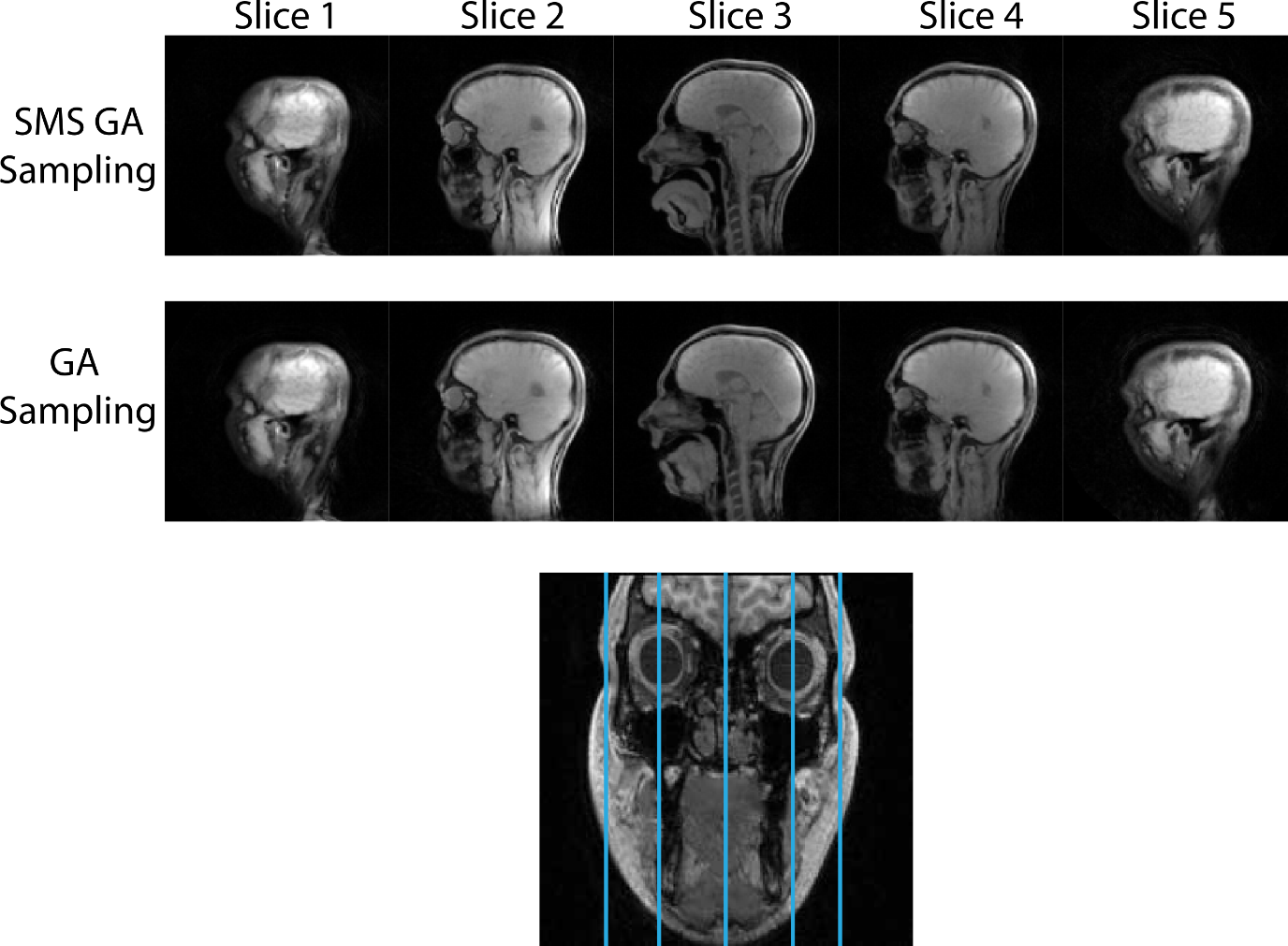


**Supplementary Figure 10:** A frame from five slice rtMRI videos acquird with SMS GA sampling (top) and GA sampling (bottom) with a slice distance of 8mm and 45 spokes/frame. The coronal image (bottom) indicates the approximate slice positions.

Real-time MRI videos at undersampling levels of 45, 25 and 15 spokes/frame were reconstructed (Supplementary Video 9). Figure S11 compares three frames, reconstructed using 15 spokes/frame, acquired using GA sampling and SMS GA sampling and, in both cases, significant blurring and artefacts are visible. The tSNR images (Figure S14 (top)) show that the artefacts in the GA sampling appear to have more structure compared to the SMS GA sampling scheme.


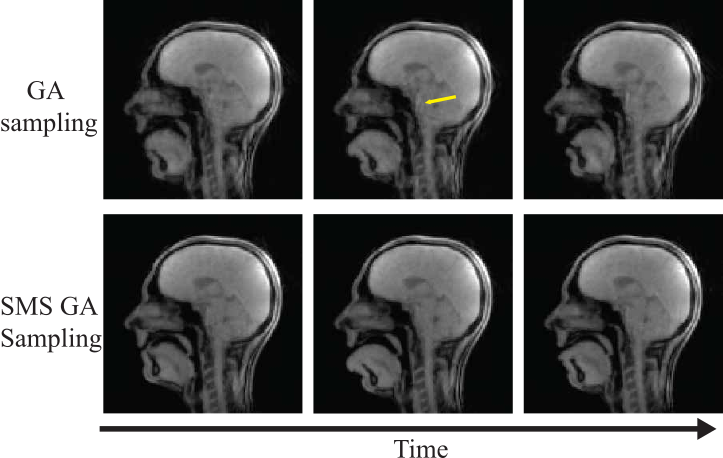


**Supplementary Figure 11:** Comparison of three frames (15 spokes/frame) acquired using the GA sampling scheme (top) and SMS GA sampling scheme (bottom). The central slice from a 5-slice acquisition is shown. Blurring and artefacts are present in both sampling schemes. An example of one of these artefacts is indicated by the yellow arrow.

Increasing the number of spokes/frame to 25 suppresses the most severe artefacts at the cost of reducing temporal resolution from 37.5ms to 62.5ms (Figure S12). From the tSNR images (Figure S14 (middle)), the artefacts seen when using GA sampling have been reduced. The overall tSNR is higher for GA SMS compared to SMS GA sampling, this may be caused by the reduced k-space coverage of SMS GA sampling.


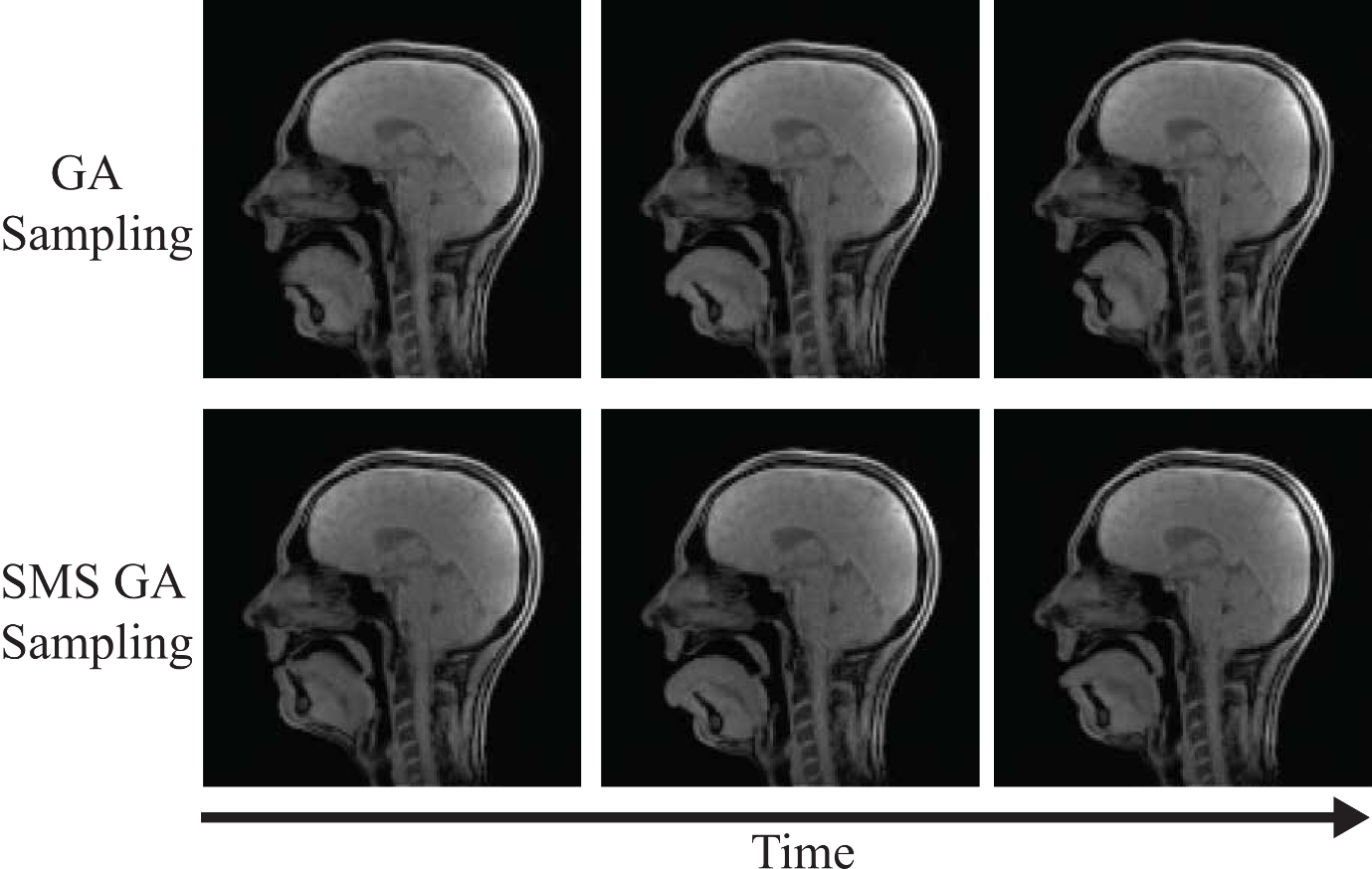


**Supplementary Figure 12:** Comparison of three frames (25 spokes/frame) acquired using the GA sampling scheme (top) and SMS GA sampling scheme (bottom). The central slice from a 5-slice acquisition is shown.

Further increasing the number of spokes to 45 results in rtMRI videos which do not display large intensity variations (Figure S13). This reduction in intensity variation is reflected in the tSNR images (Figure S14 (bottom)).


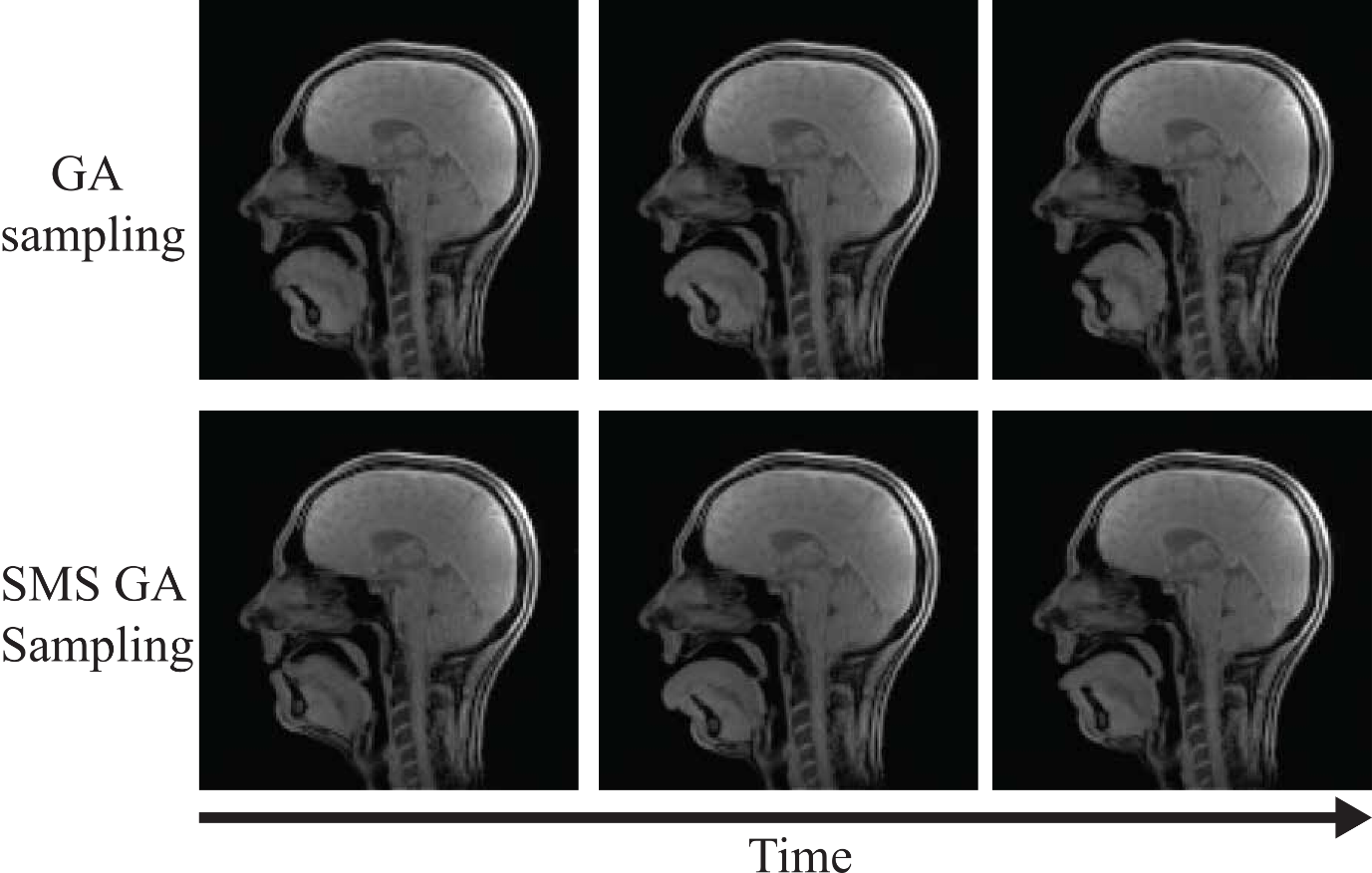


**Supplementary Figure 13:** Comparison of three frames (25 spokes/frame) acquired using the GA sampling scheme (top) and SMS GA sampling scheme (bottom). The central slice from a 5-slice acquisition is shown.


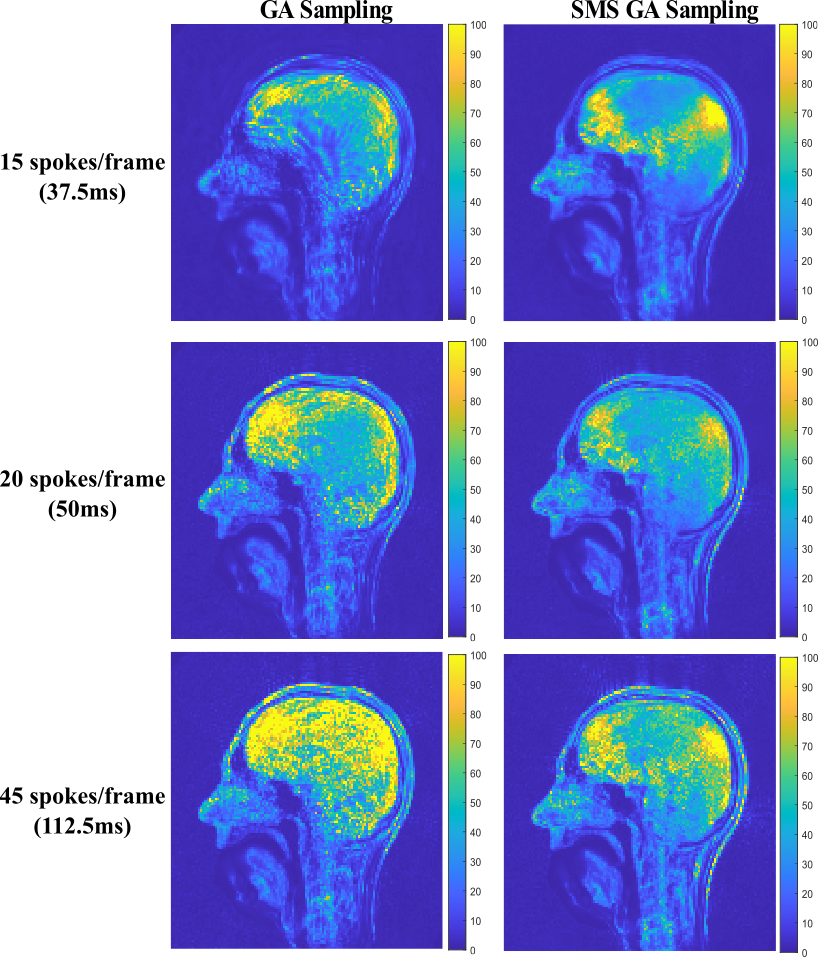


**Supplementary Figure 14:** tSNR comparison of GA sampling and SMS GA sampling at different levels of undersampling. This shows the degradation in tSNR, for both sampling schemes, as the number of spokes/frame is decreased.
